# P-Rex1 limits the agonist-induced internalization of GPCRs independently of its Rac-GEF activity

**DOI:** 10.1016/j.celrep.2025.116403

**Published:** 2025-10-14

**Authors:** Martin J. Baker, Elizabeth Hampson, Priota Islam, Ruben Pelaez Moral, Eve A. Maunders, Kirsti Hornigold, Elpida Tsonou, Angeliki Malliri, David C. Hornigold, Roderick E. Hubbard, Andrew J. Massey, Heidi C.E. Welch

**Affiliations:** 1 Signalling Programme, The Babraham Institute, Babraham Research Campus, Cambridge, UK; 2 Cell Signalling Group, Cancer Research UK Manchester Institute, University of Manchester, Wilmslow Road, Manchester, UK; 3 Bioscience Metabolism, Research and Early Development, Cardiovascular, Renal and Metabolism (CVRM), BioPharmaceuticals R&D, AstraZeneca, Cambridge, UK; 4 Vernalis (R&D) Ltd., Granta Park, Cambridge, UK

**Keywords:** PREX1, PREX2, guanine-nucleotide exchange factor, GEF, G protein-coupled receptor, GPCR, agonist-induced internalization, trafficking, GRK2

## Abstract

The guanine-nucleotide exchange factor (GEF) P-Rex1 mediates G protein-coupled receptor (GPCR) signaling by activating the small GTPase Rac. We show here that P-Rex1 also controls GPCR trafficking. P-Rex1 inhibits the agonist-stimulated internalization of the GPCR S1PR1 independently of its Rac-GEF activity, through its PDZ, DEP, and inositol polyphosphate 4-phosphatase domains. P-Rex1 also limits the agonist-induced trafficking of CXCR4, PAR4, and GLP1R but does not control steady-state GPCR levels, nor the agonist-induced internalization of the receptor tyrosine kinases PDGFR and EGFR. P-Rex1 blocks the phosphorylation required for GPCR internalization. P-Rex1 binds G protein-coupled receptor kinase 2 (Grk2), both *in vitro* and in cells, but does not appear to regulate Grk2 activity. We propose that P-Rex1 limits the agonist-induced internalization of GPCRs through its interaction with Grk2 to maintain high levels of active GPCRs at the plasma membrane. Therefore, P-Rex1 plays a dual role in promoting GPCR responses by controlling GPCR trafficking through an adapter function as well as by mediating GPCR signaling through its Rac-GEF activity.

## Highlights

• P-Rex1 controls GPCR trafficking, independently of its Rac-GEF activity
• P-Rex1 limits the agonist-induced internalization of S1PR1, CXCR4, PAR4, and GLP1R
• P-Rex1 does not control steady-state GPCR levels, nor PDGFR or EGFR trafficking
• P-Rex1 binds Grk2 and inhibits the phosphorylation required for GPCR internalization

## Graphical abstract

**Figure:**
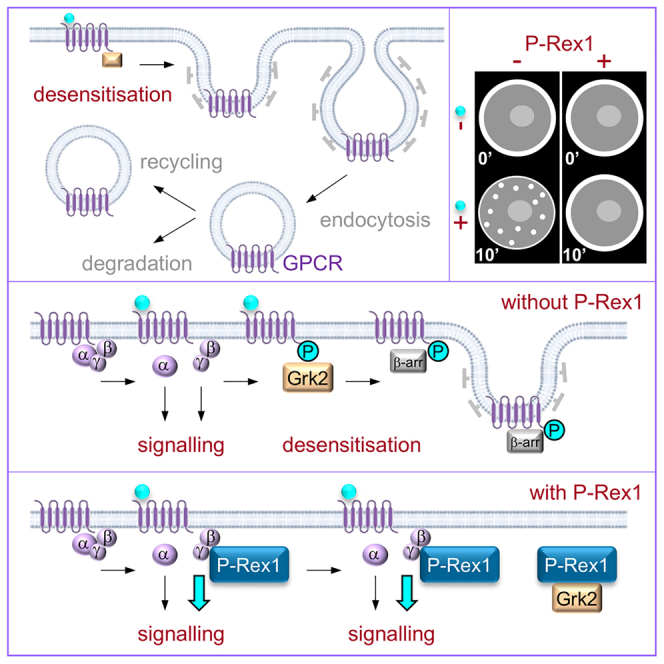

## Teaser

P-Rex1 activates Rac downstream of GPCRs to regulate processes ranging from innate immunity to neuronal plasticity, its deregulation contributing to cancer. Here, Baker et al. show that P-Rex1 also controls GPCR trafficking, limiting agonist-induced GPCR internalization through an adapter function. Thus, P-Rex1 promotes GPCR responses in a dual manner.

## Introduction

P-Rex1 is a widely expressed guanine nucleotide exchange factor (GEF) for Rac-type small GTPases, which plays important roles in the immune and nervous systems.^1^^,^^2^^,^^3^^,^^4^ In the immune system, P-Rex1 mediates pro-inflammatory and immune functions, including leukocyte recruitment and the clearance of bacteria.^3^^,^^5^^,^^6^^,^^7^^,^^8^ In the nervous system, P-Rex1 modulates synaptic plasticity, fine motor skills, and social recognition.^9^^,^^10^^,^^11^ Furthermore, P-Rex1 is required for melanoblast migration during development, controlling skin pigmentation.^12^ Deregulation of P-Rex1 levels contributes to diseases. P-Rex1 overexpression is seen in many types of cancers, including breast cancer and melanoma, promoting tumor initiation, growth, and/or invasiveness.^12^^,^^13^^,^^14^^,^^15^^,^^16^^,^^17^^,^^18^^,^^19^ Loss of P-Rex1 is associated with autism spectrum disorders.^11^ In mice, P-Rex1 promotes pulmonary fibrosis^20^ and diet-induced non-alcoholic fatty liver disease.^21^

All physiological and pathophysiological functions of P-Rex1 are either known or assumed to be mediated through its catalytic Rac-GEF activity. P-Rex1 activates Rac isoforms (Rac1, Rac2, Rac3, and RhoG) upon synergistic activation by the phosphoinositide 3-kinase (PI3K)-generated lipid second messenger phosphoinositide 3,4,5-trisphosphate (PIP_3_) and the Gβγ subunits of heterotrimeric G proteins released upon the activation of G protein-coupled receptors (GPCRs).^1^ P-Rex1 has an N-terminal catalytic Dbl homology (DH) domain, which serves to activate Rac, a Pleckstrin homology (PH) domain, two Disheveled, Egl-10, and Pleckstrin (DEP) domains, and two PSD-95, Dlg, and ZO-1 (PDZ) domains, and a C-terminal half similar to inositol polyphosphate 4-phosphatase (IP4P) but without phosphatase activity.^1^^,^^3^ Residues E56 and N238 in the DH domain are required for the interaction with Rac1 and catalytic activity.^4^^,^^22^ PIP_3_ binds to the PH domain, and Gβγ binds to the DH domain and C-terminal domains, whereas the DEP, PDZ, and IP4P domains keep the protein in an autoinhibited conformation prior to cell stimulation.^22^^,^^23^^,^^24^^,^^25^^,^^26^^,^^27^^,^^28^^,^^29^^,^^30^

In addition to P-Rex1, the P-Rex family comprises P-Rex2, which shares the domain structure and regulation by PIP_3_ and Gβγ.^9^^,^^31^^,^^32^ Unlike P-Rex1, P-Rex2 has a known adaptor function, inhibiting the tumor suppressor phosphatase and tensin homolog deleted on chromosome 10 (PTEN), which converts PIP_3_ to PI(4,5)P_2_, and thus indirectly stimulating the PI3K pathway activity.^33^^,^^34^^,^^35^ This adaptor function is specific to P-Rex2, as P-Rex1 cannot bind PTEN.^33^

GPCRs are the largest family of cell surface receptors, characterized by their seven transmembrane spanning regions.^36^ They signal in response to a vast array of stimuli, ranging from photons to proteins, peptides, and lipids.^36^ Ligand binding induces a conformational change, which activates the receptor-coupled heterotrimeric G protein, consisting of Gα and Gβγ.^37^ The Gα subunit is a GTPase, and the ligand-bound GPCR acts as its GEF, leading to GTP-loading of Gα and release of the Gβγ dimer. Gα-GTP and Gβγ then interact with their respective effector proteins to elicit downstream signaling.^37^

GPCRs are classified by the Gα they couple to: Gα_s_, Gα_i_, Gα_q_, or Gα_12/13_. Generally, Gα_s_ signals to increase adenylyl cyclase activity and cellular cyclic AMP levels, Gα_i_ inhibits adenylyl cyclase, Gα_q_ activates phospholipase-C-β (PLCβ) to stimulate Ca^2+^ signaling, and Gα_12/13_ activates Rho-GEFs to regulate cytoskeletal organization.^38^ However, all Gα proteins signal through multiple pathways. Like Gα, Gβγ proteins also regulate various signaling pathways, including the P-Rex, PLCβ, and PI3Kγ pathways.^39^

Desensitization of activated GPCRs occurs largely through agonist-induced internalization.^40^^,^^41^ G protein-coupled receptor kinases (Grks) phosphorylate serine or threonine residues in the cytoplasmic C-terminal tail of active GPCRs. β-arrestin is recruited to the phosphorylated receptor, which recruits clathrin adaptor AP2, Arf-GTPase Arf6, and clathrin, leading to clathrin-mediated endocytosis, as well as sterically hindering the coupling between GPCR and heterotrimeric G protein in some cases. This usually terminates the GPCR signal, although some internalized GPCRs continue to signal intracellularly. The internalized GPCRs are either recycled back to the cell membrane following dephosphorylation or transported to lysosomes for degradation.^40^^,^^41^

The Grk family comprises seven members.^42^ Grk2, the ubiquitously expressed prototype, carries an N-terminal regulator of G proteins signaling homology (RH) domain, the central catalytic domain, and the C-terminal PH domain.^43^^,^^44^ Binding of Gβγ to the PH domain upon GPCR activation recruits Grk2 to the plasma membrane, enabling GPCR phosphorylation.^42^

Here, we investigated the roles of P-Rex1 in GPCR trafficking and show that P-Rex1 binds directly to Grk2 and limits the agonist-induced internalization of GPCRs through an adaptor function.

## Results

### P-Rex1 limits the agonist-induced internalization of the GPCR S1PR1 independently of its catalytic Rac-GEF activity

Sphingosine 1-phosphate receptor 1 (S1PR1) is a widely expressed GPCR for sphingosine 1-phosphate (S1P), with essential roles in vascular and neuronal development and pleiotropic other functions.^45^ Upon S1P stimulation, S1PR1 signals through to Gα_i_ and is then internalized by clathrin-mediated endocytosis to switch off its signaling,^46^^,^^47^^,^^48^ although the internalized receptor can continue to signal after stimulation with certain other, engineered agonists.^49^ S1PR1 internalization occurs upon phosphorylation by Grk2 and in a β-arrestin-dependent manner.^47^^,^^48^ To study S1PR1 trafficking, we generated HEK293 cells which stably express S1PR1-GFP (HEK293-S1PR1 cells), similar to a neuronal PC12-S1PR1 cell line we established previously.^50^^,^^51^ Widefield fluorescence microscopy showed that S1PR1-GFP is localized at the plasma membrane of serum-starved cells, as judged by the sheet-like staining typically seen in widefield imaging,^52^ and is internalized into vesicles upon S1P stimulation in a dose- and time-dependent manner, with a half-maximal effective concentration (EC_50_) of 5 nM S1P after 30 min and with 50% internalization after 17 min in response to 10 nM S1P (Figures S1A–S1D). Confocal live imaging in the presence of CellMask Orange confirmed that S1PR1-GFP is internalized from the plasma membrane into vesicles upon S1P stimulation (Supplemental Video 1 and Figure S2A). Use of the small-molecule inhibitor Pitstop2 confirmed that S1PR1-GFP is internalized via clathrin-mediated endocytosis (Figure S2B), and use of the pan-Grk inhibitor CCG215022 confirmed that Grk-mediated phosphorylation is required for the internalization of the receptor (Figure S2C). We assessed the localization of S1PR1-GFP by two image analysis methods: a semi-quantitative method comparing the images to a panel of standard images (Figures S1A, S1B, and S1E) and a quantitative method using Volocity image analysis^51^ (Figures S3A–S3C), which gave similar results.

Video S1. Confocal fluorescence microscopy showing that S1PR1-GFP is internalized from the plasma membrane of serum-starved HEK293-S1PR1 cells stained with CellMask Orange into vesicles during stimulation with 1 nM S1P over the course of 30 min

**Figure 1:**
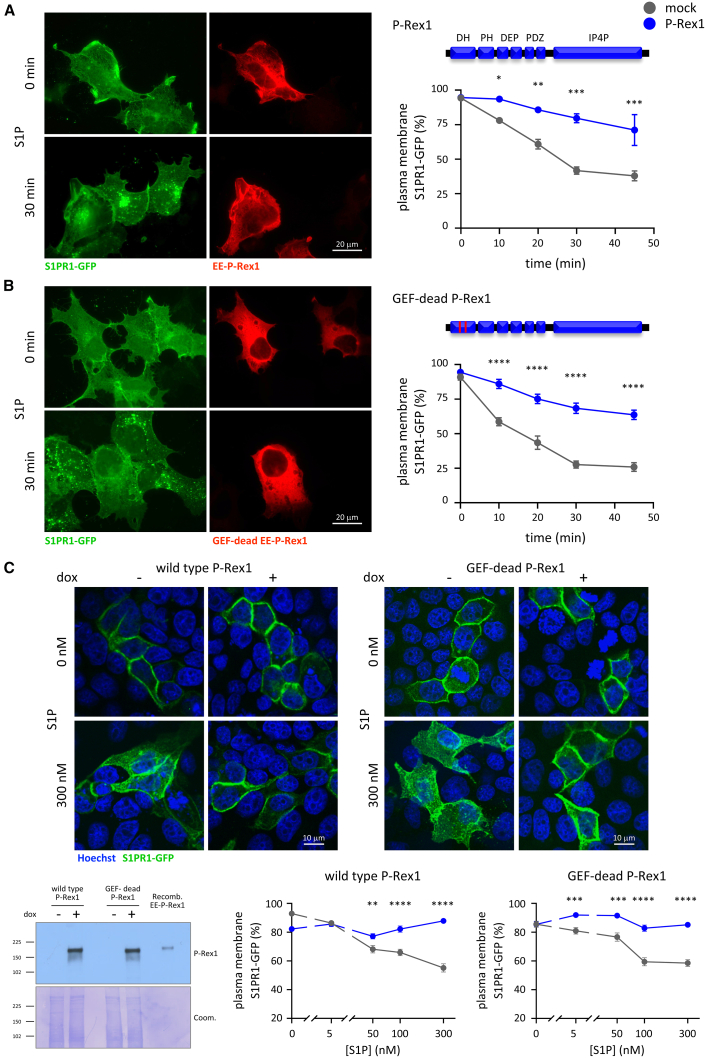
P-Rex1 limits the S1P-stimulated internalization of S1PR1 independently of its catalytic Rac-GEF activity (A and B) Wild-type and GEF-dead P-Rex1 limit the S1P-induced internalization of S1PR1. HEK293-S1PR1 cells, stably expressing S1PR1-GFP, were transiently transfected with wild-type (A) or GEF-dead (B) EE-P-Rex1 (blue symbols), or mock transfected (gray symbols), serum starved for 6.5 h, stimulated with 10 nM S1P for the indicated periods of time, fixed, stained for EE (red), and analyzed by widefield microscopy with focus on the apical cell surface. Representative images show cells stimulated for 0 and 30 min, respectively. S1PR1-GFP localization at the plasma membrane was quantified by blinded comparison to standard widefield images (Figures S1A and S1B). Alternative quantification of the same data by Volocity image analysis is shown in Figures S3B and S3C. Data are mean ± SEM of three independent experiments. Statistics are two-way ANOVA with Sidak’s multiple comparisons correction; stars denote differences between genotypes for each time point; ^∗^ indicates *p* < 0.05, ^∗∗^*p* < 0.01, ^∗∗∗^*p* < 0.001, and ^∗∗∗∗^*p* < 0.0001. (C) Inducible expression of wild-type or GEF-dead P-Rex1 inhibits the S1P-stimulated internalization of S1PR1. MDCK cells with doxycycline (dox)-inducible expression of wild-type or GEF-dead P-Rex1 were treated with (blue lines) or without (gray lines) 1 μg/mL dox for 24 h, serum starved for 18 h, and then stimulated with the indicated concentrations of S1P for 10 min, fixed, stained with Hoechst 33342, and imaged by confocal fluorescence microscopy with focus on a central plane. Representative confocal images are shown. Quantification was done as in (A) and (B), except that blinded images were compared to standard confocal images (Figure S1E). Data are mean ± SEM of 66–95 cells per condition. Statistics are two-way ANOVA with Sidak’s multiple comparisons correction; stars denote differences between genotypes for each S1P concentration. The western blot shows the dox-induced expression of wild-type or GEF-dead P-Rex1 under the same conditions. Recombinant EE-P-Rex1 was loaded as a control. Coomassie staining was used to control for protein loading.

To investigate whether P-Rex1 controls S1PR1 trafficking, we expressed EE-tagged P-Rex1 transiently in HEK293-S1PR1 cells, stimulated the cells with 10 nM S1P, fixed them, and imaged them for EE-P-Rex1, and the localization of S1PR1-GFP. EE-P-Rex1 expression limited the S1P-stimulated internalization of the receptor (Figure 1A). The same results were obtained by live-cell imaging. HEK293-S1PR1 cells showed robust internalization of S1PR1-GFP from the plasma membrane into intracellular vesicles in response to S1P (Supplemental Video 2), whereas mCherry-P-Rex1 reduced this internalization (Supplemental Video 3). Therefore, P-Rex1 limits the agonist-induced internalization of the GPCR S1PR1. In contrast, S1PR1 localization was normal without S1P stimulation (Figures 1A and S3B), suggesting that P-Rex1 inhibits agonist-dependent but not steady-state receptor trafficking. Furthermore, P-Rex1 did not affect the total expression level of the GPCR (Figure S3D).

Video S2. Widefield fluorescence microscopy showing that S1PR1-GFP is internalized in serum-starved HEK293-S1PR1 cells during stimulation with 100 nM S1P (added at the flash) over the course of 45 min

Video S3. Widefield fluorescence microscopy showing that expression of mCherry-P-Rex1 inhibits the internalization of S1PR1-GFP in serum-starved HEK293-S1PR1 cells during stimulation with 100 nM S1P (added at the flash) over the course of 45 min

To determine if suppression of GPCR trafficking depends on the catalytic activity of P-Rex1, we expressed GEF-dead P-Rex1, which contains point mutations E56A and N238A in the DH domain that abolish Rac-GEF activity.^22^^,^^25^ As for wild-type P-Rex1, S1PR1-GFP was retained at the plasma membrane of GEF-dead EE-P-Rex1 expressing cells stimulated with S1P (Figures 1B and S3C). Hence, the P-Rex1 control of GPCR trafficking is independent of its catalytic Rac-GEF activity.

To test different cells, we used MDCK cells with doxycycline (dox)-inducible expression of wild-type or GEF-dead P-Rex1^22^^,^^53^ (Figure 1C), together with transient expression of S1PR1-GFP, and performed confocal fluorescence microscopy. Without dox-induction, S1PR1-GFP appeared as a ring at the cell periphery, as typical for plasma membrane-localized proteins by confocal microscopy,^52^ and was dose-dependently internalized into intracellular vesicles upon S1P stimulation. The expression of wild-type or GEF-dead P-Rex1 inhibited this internalization (Figure 1C).

Therefore, P-Rex1 inhibits the agonist-induced internalization of S1PR1-GFP independently of its catalytic Rac-GEF activity. This is the first Rac-GEF activity-independent function of P-Rex1.

### P-Rex1 deficiency increases the agonist-induced internalization of S1PR1

**Figure 2:**
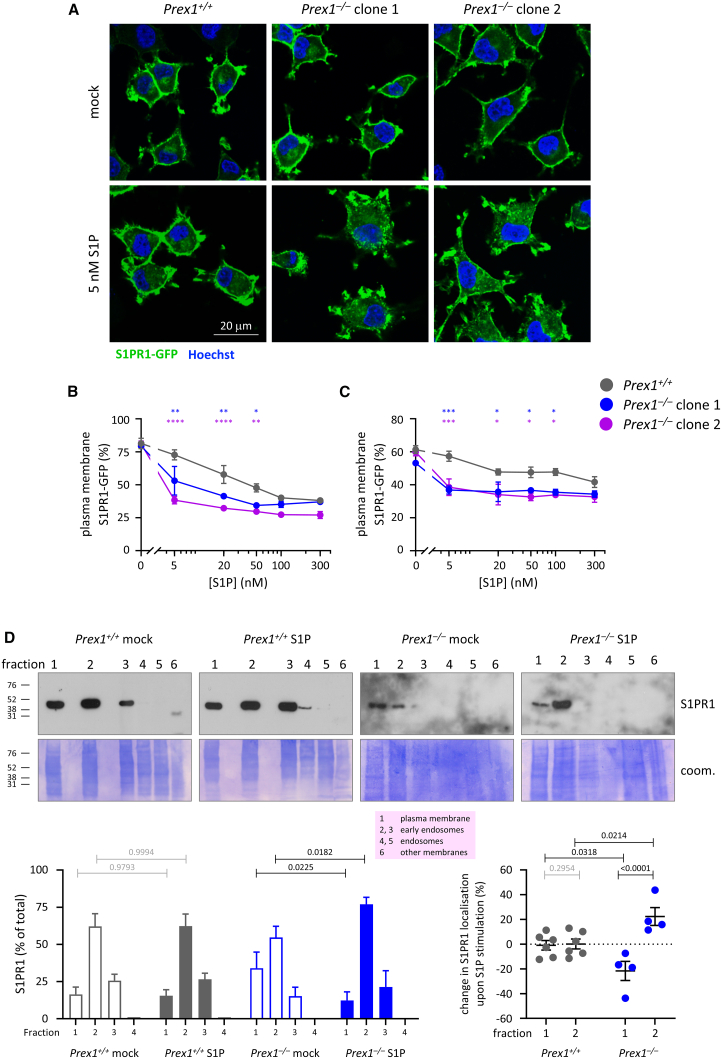
P-Rex1 deficiency promotes the S1P-dependent internalization of S1PR1 (A–C) Wild-type PC12-S1PR1 cells (*Prex1*^*+/+*^, gray symbols) and P-Rex1-deficient (*Prex1*^−/−^) PC12-S1PR1 cells clone 1 (blue) and clone 2 (purple) were serum starved for 14 h, stimulated with the indicated concentrations of S1P for 10 min, fixed, stained with Hoechst 33342, and imaged by confocal microscopy with focus on a central plane. (A) Representative confocal images are shown. S1PR1-GFP localization at the plasma membrane was quantified by (B) blinded comparison to standard images (Figure S1E) or (C) CellProfiler analysis (Figure S3A). Data in (B) and (C) are mean ± SEM of ≥3 independent experiments; with ≥100 cells analyzed for each genotype and condition per experiment; the same experiments were analyzed by both methods. Statistics are two-way ANOVA with Sidak’s multiple comparisons correction; stars denote differences between genotypes for each S1P concentration; and ^∗^ indicates *p* < 0.05, ^∗∗^*p* < 0.01, ^∗∗∗^*p* < 0.001, and ^∗∗∗∗^*p* < 0.0001. (D) Fractionation of PC12-S1PR1 cells. *Prex1*^*+/+*^ (gray) and *Prex1*^−/−^ (blue) PC12-S1PR1 cells were serum starved for 14 h and stimulated with 5 nM S1P or mock stimulated for 10 min. Detergent-free cell lysates were fractionated by discontinuous OptiPrep density gradient (see Figure S4), and proteins from each fraction analyzed by western blotting with S1PR1 antibody. All of fractions 1–5 and 50% of fraction 6 were loaded. Representative western blots are shown. Coomassie staining was used to show total protein. Lower left: the amount of endogenous S1PR1 per fraction was quantified by Fiji densitometry. Lower right: change in S1PR1 localization in fractions 1 and 2 upon S1P stimulation. Data are mean ± SEM from 4 to 6 independent experiments. Statistics are two-way ANOVA with Sidak’s multiple comparisons correction; black *p* values denote significant differences, and gray *p* values are not significant.

To investigate if endogenous P-Rex1 controls the S1P-stimulated internalization of S1PR1-GFP, we used wild-type (*Prex1*^*+/+*^) and two clones of P-Rex1-deficient (*Prex1*^−/−^) PC12-S1PR1 cells.^50^ The cells were serum-starved, stimulated with S1P, and the localization of S1PR1-GFP was assessed by confocal microscopy (Figures 2A–2C and S1E). P-Rex1 deficiency increased S1P-induced receptor internalization, whereas the steady state-cell surface receptor level was normal (Figures 2B and 2C). Hence, P-Rex1 expression limits and P-Rex1 deficiency promotes the agonist-induced internalization of S1PR1-GFP.

To assess if P-Rex1 controls the trafficking of endogenous S1PR1, and for an alternative method of quantifying receptor internalization, we adapted a cell fractionation method separating endosomes from plasma membrane^54^ using ultracentrifugation of detergent-free PC12-S1PR1 cell lysates in an OptiPrep density gradient followed by western blotting. This provided a K-Ras-enriched plasma-membrane fraction (fraction 1), EEA1-enriched early endosome fractions (2, 3), and Rab5-enriched endosome fraction (5) (Figures S4A–S4C). Fractionation of serum-starved *Prex1*^*+/+*^ and *Prex1*^−/−^ PC12-S1PR1 cells showed that endogenous S1PR1 was mainly localized in the plasma membrane and early endosome fractions. Upon stimulation with a low concentration of S1P (5 nM), 22% of cellular S1PR1 translocated from the plasma membrane into the early endosome fraction in *Prex1*^−/−^ PC12-S1PR1 cells, whereas receptor localization remained unchanged in *Prex1*^*+/+*^ PC12-S1PR1 cells (Figure 2D). Therefore, P-Rex1 limits the agonist-induced internalization of endogenous S1PR1. The extent of internalization of endogenous S1PR1 measured by fractionation was identical to that of S1PR1-GFP assessed by imaging at that concentration of S1P. To test the specificity of S1PR1 internalization, we also determined the localization of the receptor tyrosine kinase (RTK) EGFR. Endogenous EGFR was localized throughout the plasma membrane and endosomal fractions in both *Prex1*^*+/+*^ and *Prex1*^−/−^ PC12-S1PR1 cells, and S1P stimulation did not affect it (Figure S4D).

### Large portions of the P-Rex1 protein are required for its control of GPCR trafficking

**Figure 3:**
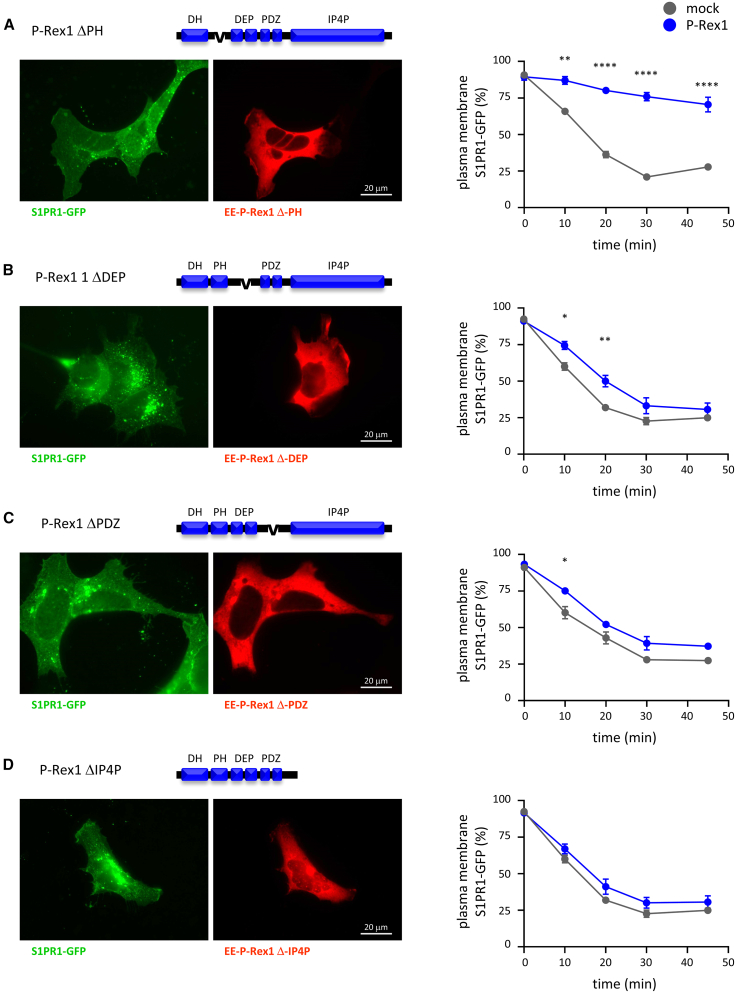
The DEP, PDZ, and IP4P domains of P-Rex1 are required for the inhibition of S1PR1 internalization HEK293-S1PR1 cells were transfected with (A) EE-P P-Rex1 ΔPH, (B) EE-P P-Rex1 ΔDEP, (C) EE-P P-Rex1 ΔPDZ, or (D) EE-P-Rex1 ΔIP4P (blue symbols), or mock transfected (gray symbols), serum starved for 6.5 h, stimulated with 10 nM S1P for the indicated periods of time, fixed, stained for EE (red), and analyzed by widefield microscopy with focus on the apical cell surface. Representative images show cells stimulated with S1P for 30 min. S1PR1-GFP localization at the plasma membrane was quantified by comparison to standard images (Figures S1A and S1B). Alternative quantification of the same data by Volocity image analysis is shown in Figure S5. Data are mean ± SEM of three independent experiments for each mutant; statistics are two-way ANOVA with Sidak’s multiple comparisons correction; stars denote differences between cells with and without P-Rex1 mutant for each time point. ^∗^*p* < 0.05, ^∗∗^*p* < 0.01, ^∗∗∗^*p* < 0.001, and ^∗∗∗∗^*p* < 0.0001.

To identify which P-Rex1 domains are required for controlling GPCR trafficking, we transiently expressed various P-Rex1 mutants.^22^^,^^55^ Western blotting showed that all P-Rex1 mutants expressed at a similar level in their transiently transfected cell populations, except ΔPDZ, which was lower (Figure S5A), and we chose cells with comparable expression levels for blinded image analysis (Figure 3). EE-P-Rex1 ΔPH inhibited the S1P-induced internalization of S1PR1-GFP, like the wild-type P-Rex1 (Figures 3A and S5B), suggesting that the PH domain is dispensable. In contrast, EE-P-Rex1 ΔPDZ, ΔDEP, and ΔIP4P mutants had little or no effect on S1PR1-GFP trafficking (Figures 3B–3D and S5C–S5E). Therefore, large portions of the P-Rex1 protein, including the DEP, PDZ, and IP4P domains, are required to inhibit GPCR trafficking. Furthermore, it was previously suggested that the isolated PDZ domains of P-Rex1 can interact with S1PR1.^56^ To investigate, we expressed myc-P-Rex1 iPDZ in HEK293-S1PR1 cells. This had no effect on S1PR1-GFP trafficking (Figure S6A), suggesting that the isolated PDZ-domain tandem is not sufficient for controlling S1PR1 trafficking.

To investigate if the P-Rex1 homolog P-Rex2 plays a similar role, we expressed myc-P-Rex2 in HEK293-S1PR1 cells. P-Rex2 had a similar effect on S1P-induced S1PR1-GFP trafficking to P-Rex1 (Figure S6B). Therefore, both P-Rex family members limit agonist-induced GPCR internalization.

### P-Rex1 limits the agonist-induced internalization of a range of GPCRs but not RTKs

To investigate if other GPCRs are affected by P-Rex1, in addition to S1PR1, we selected candidate GPCRs to cover a range of classes coupling to different types of heterotrimeric G protein and expressed them in MDCK cells with dox-inducible expression of wild-type or GEF-dead P-Rex1. The GPCRs included Gα_i_-coupled CXCR4,^57^ Gα_s_-coupled glucagon-like peptide-1 receptor (GLP1R),^58^ and Gα_q/12/13_-coupled protease-activated receptor 4 (PAR4).^59^ Each GPCR was assessed by confocal fluorescence microscopy, using stimulation with its own agonist, after pilot experiments to determine appropriate agonist concentrations and timing.

**Figure 4:**
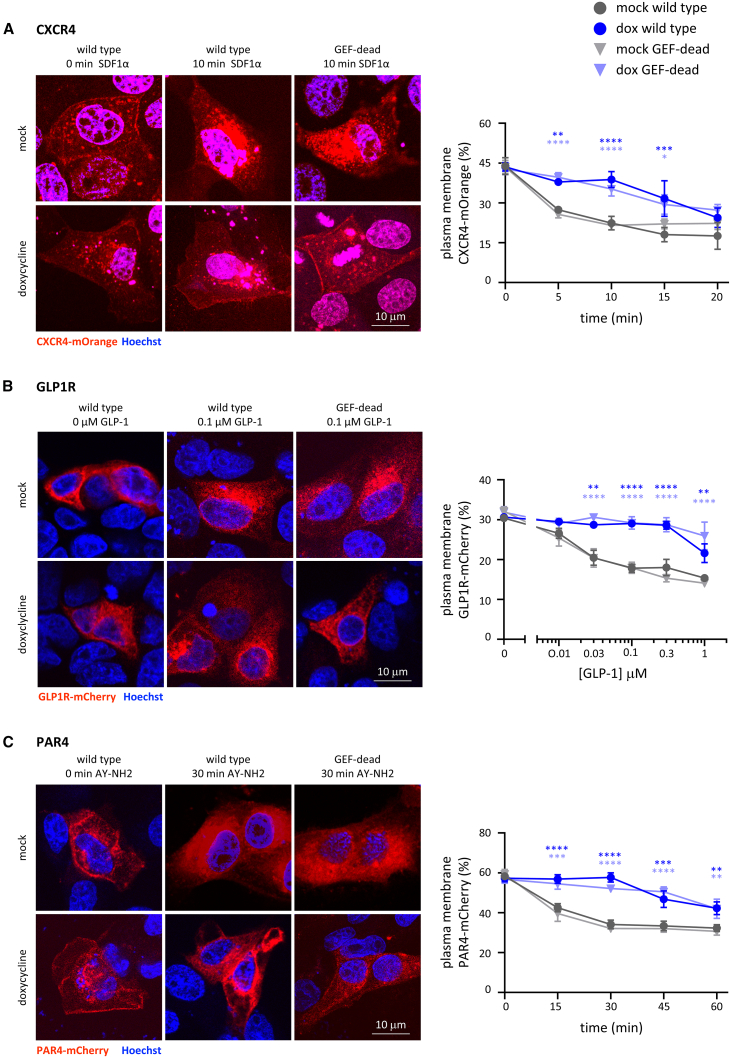
Expression of P-Rex1 inhibits the agonist-induced internalization of CXCR4, GLP1R, and PAR1, independently of its catalytic Rac-GEF activity (A) CXCR4. CXCR4-LSSmOrange was expressed in MDCK cells with dox-inducible wild-type (circles) or GEF-dead (triangles) P-Rex1. Cells were treated with 1 μg/mL dox (blue symbols) for 24 h or mock treated (gray symbols), serum starved, stimulated with 25 nM SDF1α for the indicated periods of time, fixed, stained with Hoechst 33342, and imaged by confocal fluorescence microscopy with focus on a central plane. Representative confocal images are shown. CXCR4-LSSmOrange localization at the plasma membrane was quantified by comparison to standard images (Figure S1E). (B) GLP1R. MDCK cells were treated as in (A) except that GLP1R-mCherry was expressed and cells were stimulated with the indicated concentrations of GLP-1 for 10 min. GLP1R-mCherry localization was quantified as in (A). (C) PAR4. MDCK cells were treated as in (A) and (B) except that PAR4-mCherry was expressed and cells were stimulated with 500 μM AY-NH2 for the indicated periods of time. PAR4-mCherry localization was quantified as in (A). Data in (A)–(C) are mean ± SEM of three independent experiments for each receptor. Statistics are two-way ANOVA with Sidak’s multiple comparisons correction; stars denote differences between mock and dox conditions.

MDCK cells expressing CXCR4-LSSmOrange were treated with dox to induce P-Rex1 expression, or mock-treated, then serum-starved, and stimulated with 25 nM stromal cell-derived factor-1α (SDF1α). The localization of CXCR4-LSSmOrange was more cytoplasmic than observed for S1PR1, even partially nuclear, which had been observed before,^60^ but SDF1α stimulation induced the expected dose-dependent internalization from the plasma membrane into intracellular vesicles. Both wild-type and GEF-dead P-Rex1 inhibited this SDF1α-stimulated internalization of CXCR4-LSSmOrange (Figure 4A). Similarly, MDCK cells expressing GLP1R-mCherry were stimulated with various concentrations of glucagon-like peptide-1 (GLP-1).^61^ Again, the localization of GLP1R-mCherry was more cytoplasmic than observed for S1PR1, but GLP-1 stimulation caused the expected dose-dependent internalization of the receptor, and both wild-type and GEF-dead P-Rex1 inhibited this internalization (Figure 4B). MDCK cells expressing PAR4-mCherry were stimulated with 500 μM AY-NH2,^62^ which caused receptor internalization, and again, both wild-type and GEF-dead P-Rex1 inhibited this internalization (Figure 4C). Hence, P-Rex1 inhibits the agonist-induced internalization of all GPCRs tested, regardless of the type of heterotrimeric G protein they couple to, in a manner independent of GEF activity.

To determine if P-Rex1 controls the trafficking of other receptor classes, we tested the agonist-induced internalization of the RTKs epidermal growth factor receptor (EGFR) and platelet-derived growth factor receptor β (PDGFRβ). MDCK cells expressing EGFR-GFP were serum-starved and stimulated with 100 ng/mL EGF.^63^ EGFR-GFP was largely localized at the plasma membrane of serum-starved cells, and EGF stimulation caused the expected internalization of the receptor, but this internalization was not affected by wild-type or GEF-dead P-Rex1 (Figure S7A). Similarly, PDGFRβ-GFP was also largely localized at the plasma membrane, and stimulation with 50 ng/mL PDGF^64^ induced the internalization of the receptor, which was not affected by wild-type or GEF-dead P-Rex1 (Figure S7B). Hence, P-Rex1 controls the trafficking of a range of GPCRs, in a manner independent of GEF activity, but not RTK trafficking. Of note, we previously demonstrated normal cell surface levels of L-selectin and β2 integrins LFA1 and Mac1 in *Prex1*^−/−^ mouse neutrophils.^7^^,^^65^ Hence, P-Rex1 regulates the trafficking of GPCRs but not a range of other receptor classes.

### P-Rex1 inhibits the phosphorylation required for GPCR internalization

**Figure 5:**
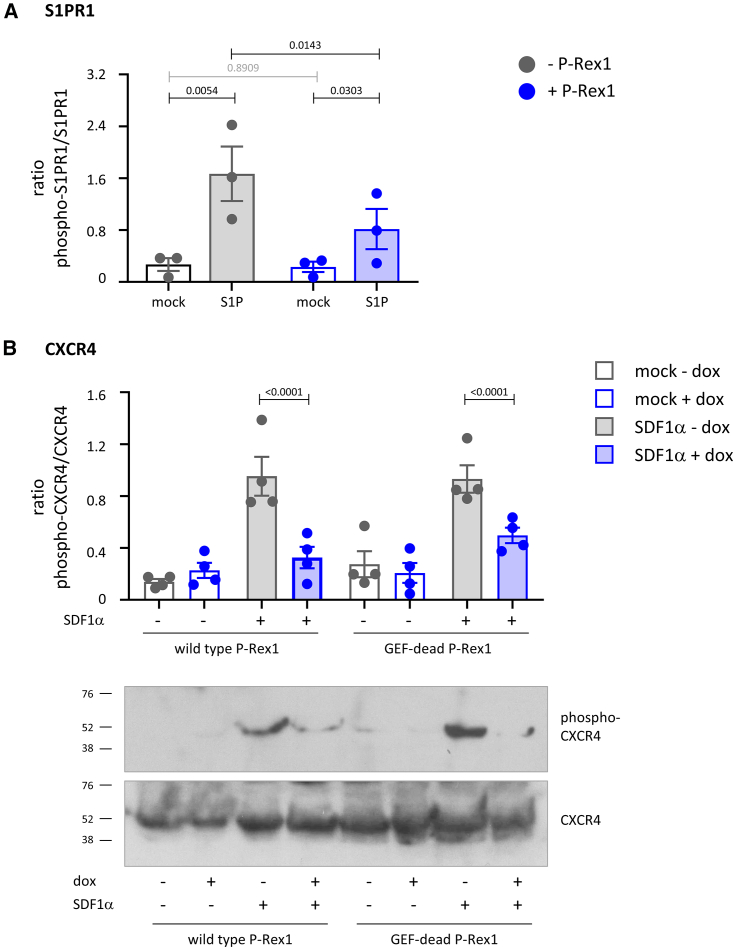
P-Rex1 inhibits the phosphorylation required for internalization of S1PR1 and CXCR4 (A) P-Rex1 inhibits the S1P-stimulated phosphorylation of S1PR1-GFP at S351. S1PR1-GFP was immunoprecipitated from lysates of HEK293-S1PR1 cells transfected with EE-P-Rex1 (blue symbols), or mock transfected (gray symbols), serum starved for 6.5 h, and stimulated with 10 nM S1P for 10 min (filled bars), or mock stimulated (open bars). Samples were analyzed by LC-MS, targeted to phosphopeptides in the S1PR1 C terminus. Data show the ratio of phosphorylated/non-phosphorylated S351 and are mean ± SEM of three independent experiments. Statistics are two-way ANOVA with Sidak’s multiple comparisons correction; black *p* values denote significant differences, and gray *p* values are not significant. (B) Wild-type and GEF-dead P-Rex1 inhibit CXCR4 phosphorylation at S3254/S325. MDCK cells were treated with 1 μg/mL dox for 24 h to induce the expression of wild-type or GEF-dead P-Rex1 (blue) or mock treated (gray), serum starved for 18 h, and stimulated with 25 nM SDF1α for 10 min (filled symbols), or mock stimulated (open symbols). Total cell lysates were western blotted with phospho-S324/S325-CXCR4 and total CXCR4 antibodies. Representative blots are shown. Blots were quantified by Fiji densitometry. Data show the ratio of phosphorylated/non-phosphorylated CXCR4 and are mean ± SEM of four independent experiments; statistics are two-way ANOVA with Sidak’s multiple comparisons correction.

The first step in agonist-induced GPCR internalization is the phosphorylation of the GPCR at its C-terminal tail, mainly by Grks.^42^ S1PR1 is trafficking is controlled by Grk2.^48^ To investigate the effects of P-Rex1 on S1PR1 phosphorylation, we used targeted mass spectrometry. HEK293-S1PR1 cells expressing EE-P-Rex1 were serum-starved and stimulated with 10 nM S1P or mock-stimulated, and S1PR1-GFP was immunoprecipitated from total lysates. Phosphorylated and non-phosphorylated peptides of the C-terminal tail of S1PR1, particularly a peptide encompassing S351, a residue critical for S1PR1 internalization, were identified via targeted liquid chromatography-mass spectrometry (LC-MS). S1P stimulation increased the phosphorylation of S351 in control cells, whereas this phosphorylation was inhibited upon P-Rex1 expression (Figure 5A). Hence, P-Rex1 inhibits the S1P-dependent phosphorylation of S1PR1 on S351, the first step in the process of clathrin-mediated receptor endocytosis.

To investigate a different GPCR and test if the blockade of GPCR phosphorylation is GEF-activity dependent, we assessed the phosphorylation of endogenous CXCR4 in dox-inducible MDCK cells. The agonist-induced phosphorylation of residues S324 and S325 by Grk2 and other kinases is required for CXCR4 internalization.^66^ MDCK cells were induced with dox or mock-treated, serum-starved, stimulated with 25 nM SDF1α, or mock-stimulated, and total lysates were western blotted for phospho-S324/S325 and total CXCR4. SDF1α stimulation increased the phosphorylation of S324/S325 in control cells, whereas expression of wild-type or GEF-dead P-Rex1 reduced this phosphorylation (Figure 5B). Hence, P-Rex1 blocks the phosphorylation required for CXCR4 internalization in a manner independent of GEF activity.

To test if P-Rex1 physically interacts with GPCRs, HEK293-S1PR1 cells expressing myc-P-Rex1, or mock-transfected, were serum-starved, stimulated with 100 nM S1P, or mock-stimulated, and total lysates were subjected to immunoprecipitation (IP) with GFP or myc antibodies using various conditions of stringency. No interaction between P-Rex1 and S1PR1-GFP could be detected under the conditions tested (Figure S8A, and data not shown).

### P-Rex1 interacts with Grk2 in cells and *in vitro*

**Figure 6:**
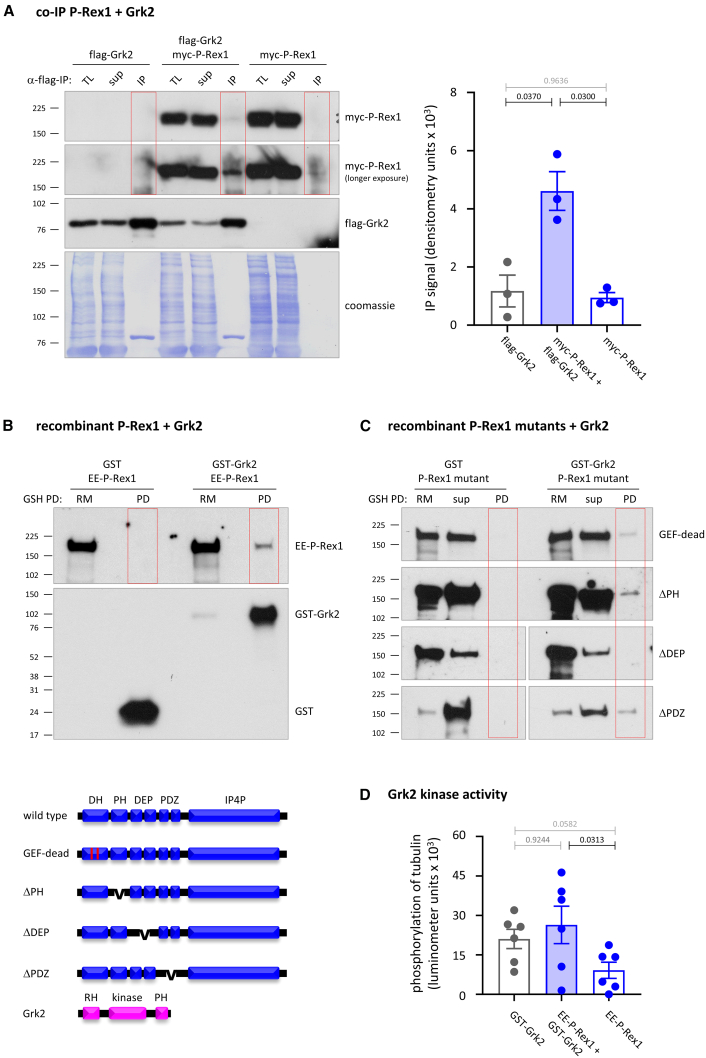
P-Rex1 interacts with Grk2 directly and in cells (A) P-Rex1 interacts with Grk2 *in vivo*. HEK293-S1PR1 cells expressing myc-P-Rex1 and/or flag-Grk2, were serum starved for 14 h, and total lysates were subjected to immunoprecipitation (IP) with flag antibody and analyzed by western blotting with myc and flag antibodies. 1.5% of the total lysate (TL) and IP supernatant (sup) were loaded alongside all the IP samples (red boxes). Coomassie staining was used as a loading control. Representative western blots are shown. Blots were quantified by Fiji densitometry. Data are mean ± SEM of three independent experiments. Statistics are one-way ANOVA with Tukey’s multiple comparisons correction; black *p* values denote significant differences, and gray *p* values are not significant. (B and C) P-Rex1 binds directly to Grk2 *in vitro* through its DEP domains. Wild-type (B) or mutant (C) EE-P-Rex1 proteins were incubated with GST or GST-Grk2, the GST-containing proteins isolated using GSH-beads, and samples western blotted with P-Rex1 and GST antibodies. 10% of the reaction mix (RM) and, where indicated, pull-down (PD) supernatant (sup) controls and all the PD samples were loaded. Blots are representative of three (B) and 2–3 (C) independent experiments per P-Rex protein. The quantification for (B) is shown in Figure S7. (D) P-Rex1 does not affect the kinase activity of Grk2. The kinase activity of GST-Grk2 was measured *in vitro*, with tubulin as the substrate, in the presence and absence of EE-P-Rex1. Data are mean ± SEM of six independent experiments. Statistics are one-way ANOVA with Tukey’s multiple comparisons correction; black *p* values denote significant differences, and gray *p* values are not significant.

To investigate if P-Rex1 interacts with Grks that phosphorylate the C terminus of GPCRs upon agonist stimulation, we selected Grk2, the best-understood Grk.^42^^,^^43^^,^^44^ To test if P-Rex1 interacts with Grk2 *in vivo*, HEK293-S1PR1 cells expressing myc-P-Rex1 and/or flag-Grk2 were serum-starved, and Grk2 was isolated from total lysates by IP with flag antibody. Myc-P-Rex1 co-immunoprecipitated with flag-Grk2 (Figure 6A), suggesting that P-Rex1 constitutively associates with Grk2 in cells.

To test if P-Rex1 binds Grk2 directly *in vitro* and determine if the interaction requires the Rac-GEF activity, we incubated purified recombinant wild-type and GEF-dead EE-P-Rex1 proteins with purified recombinant glutathione S-transferase (GST) or GST-Grk2 and isolated the GST-containing proteins by pull-down with glutathione (GSH) beads. EE-P-Rex1 bound to GST-Grk2 but not GST (Figures 6B and S8B). Hence, P-Rex1 binds Grk2 constitutively and directly. The GEF activity was not required, as GEF-dead EE-P-Rex1 also bound flag-Grk2 (Figure 6C). To test which domains of P-Rex1 are required, we used purified recombinant P-Rex1 deletion mutants.^22^^,^^55^ The PH and PDZ domains were dispensable for Grk2 binding, but the DEP domain tandem was required (Figure 6C). Hence, P-Rex1 binds Grk2 directly *in vitro* through its DEP domains, independently of its GEF activity.

To investigate which domain of Grk2 is involved in the binding, we expressed myc-P-Rex1 with either full-length flag-Grk2 or the isolated catalytic domain of flag-Grk2 (iCAT)^67^ in HEK293-S1PR1 cells and immunoprecipitated the flag-tagged proteins. Myc-P-Rex1 co-immunoprecipitated with full-length flag-Grk2, but not with the isolated catalytic domain (Figure S8C), suggesting that P-Rex1 binds the RH or PH domains of Grk2.

To test if P-Rex2 also binds Grk2, we generated human recombinant wild-type and GEF-dead His-P-Rex2 proteins, the latter by introducing alanine mutations at Glu30 and Asn212 in the catalytic DH domain, equivalent to those in GEF-dead P-Rex1,^22^^,^^55^ and purified the proteins from Sf9 cells (Figure S9). For quality control, PIP_3_-stimulated *in vitro* Rac-GEF activity assays confirmed that purified wild-type His-P-Rex2 is an active Rac-GEF and P-Rex2^E30A,N212A^ is GEF-dead (Figure S9A). Both wild-type and GEF-dead His-P-Rex2 bound to GST-Grk2 but not GST (Figure S9B). Hence, like P-Rex1, P-Rex2 binds Grk2 directly *in vitro*, independently of its GEF activity.

To test if P-Rex1 affects Grk2 activity, we measured GST-Grk2 kinase activity *in vitro*, with tubulin as the substrate. GST-Grk2 was able to phosphorylate tubulin, but EE-P-Rex1 had no effect on this kinase activity (Figure 6D). Thus, while P-Rex1 interacts with Grk2, it does not seem to control Grk2 kinase activity.

**Figure 7:**
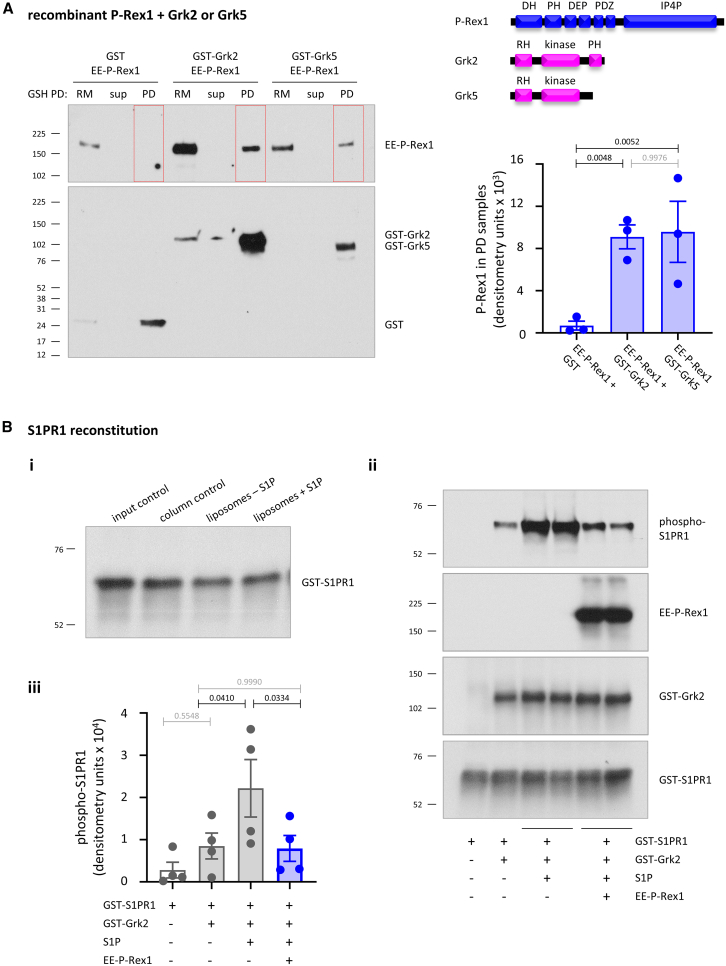
P-Rex1 interacts with Grk5 as well as Grk2 and blocks the Grk2-mediated phosphorylation of S1PR1 (A) P-Rex1 binds directly to Grk2 and Grk5 *in vitro*. GST, GST-Grk2, or GST-Grk5 were incubated with recombinant EE-P-Rex1, isolated using GSH-beads, and samples were western blotted with P-Rex1 and GST antibodies. 4% of the reaction mix control (RM), 0.4% of the pull-down (PD) supernatant control (sup), and 50% of the PD sample were loaded. Blots are from one experiment representative of three. Quantification by Fiji image analysis shows the mean ± SEM of three independent experiments. (B) GST-S1PR1 reconstitution into liposomes. Purified recombinant GST-S1PR1 was reconstituted into phosphatidylcholine liposomes by detergent withdrawal and ultracentrifugation in the presence or absence of 300 nM S1P. ∼170 nM of the reconstituted liposomal GST-S1PR1 and 100 μM ATP were incubated with or without 100 nM EE-P-Rex1, 47 nM GST-Grk2, and 300 nM S1P, and S1PR1 phosphorylation was assessed by western blotting. (i) Reconstitution efficiency. 1% of the input and post-detergent-removal column samples and of the liposomes containing reconstituted GST-S1PR1 with or without 300 nM S1P were western blotted with GST antibodies. (ii) Samples were analyzed by western blotting with antibodies to phospho-S351/S353 of S1PR1, GST (to detect GST-Grk2 and total GST-S1PR1), and P-Rex1. Blots from one representative experiment of four are shown. (iii) Quantification of S1PR1 phosphorylation by Fiji densitometry. Data are mean ± SEM of four independent experiments. Statistics in (A) and (B) are one-way ANOVA with Tukey’s multiple comparisons correction; black *p* values denote significant differences, and gray *p* values are not significant.

The pan-Grk inhibitor CCG215022 efficiently blocked the S1P-induced internalization of S1PR1-GFP in HEK293-S1PR1 cells, whereas the Grk2 inhibitor paroxetine was insufficient under the conditions tested (Figure S2C), suggesting that more than one Grk mediates S1PR1 internalization and P-Rex1 may interact with more Grks than Grk2. Therefore, we tested whether P-Rex1 can bind Grk5 as well as Grk2. Unlike Grk2, Grk5 lacks a PH domain and cannot bind Gβγ.^68^ Using recombinant EE-P-Rex1 with GST-Grk2 or GST-Grk5, we found that P-Rex1 binds directly to Grk5 as well as Grk2 (Figure 7A). Therefore, P-Rex1 binds Grks more generally. Furthermore, together with the Grk2 mutagenesis experiments in Figure S8C, these data suggest that P-Rex1 interacts with the conserved N terminus or RH domain of Grks.

Finally, after showing in Figure 6D that P-Rex1 does not affect the kinase activity of Grk2, we tested two further possible mechanisms for the P-Rex1-dependent blockade of GPCR phosphorylation by Grk. One was that Gβγ binding to P-Rex1 competes Gβγ proteins away from Gβγ-dependent Grks like Grk2, although this was not supported by P-Rex1 binding to Gβγ-independent Grk5 (Figure 7A). To investigate, we performed binding assays with recombinant EE-P-Rex1, GST-Grk2, and increasing concentrations of recombinant EE-Gβ_1_γ_2_. The Gβγ proteins partially outcompeted the binding of EE-P-Rex1 to GST-Grk2, but only at a 4-fold molar excess and had no effect at equimolar or lower concentrations (Figure S10), suggesting that Gβγ sequestration is not a major mechanism by which P-Rex1 controls GPCR trafficking.

This left the possible mechanism that P-Rex1 physically hinders the access of Grk2 to GPCRs. To test this, we adapted established protocols reconstituting functional GPCRs into liposomes^69^^,^^70^^,^^71^^,^^72^^,^^73^^,^^74^^,^^75^^,^^76^^,^^77^ for use with S1PR1. We reconstituted recombinant GST-S1PR1 into phosphatidylcholine liposomes in the presence or absence of S1P by detergent withdrawal, which resulted in the recovery of ∼50% of the GPCR in liposomes (Figure 7Bi). This liposomal GST-S1PR1 was subjected to *in vitro* phosphorylation assays using GST-Grk2, in the presence or absence of EE-P-Rex1 and S1P, followed by the assessment of S1PR1 phosphorylation using western blotting with a phospho-specific antibody for the Grk target residues S351 and S353. S1P increased S351/S353 phosphorylation by GST-Grk2, but EE-P-Rex1 blocked this phosphorylation (Figures 7Bii and iii). These data suggest that P-Rex1 controls GPCR trafficking by sequestering Grks, limiting the access of Grks to their target GPCRs.

Together, our data show that P-Rex1 limits the agonist-induced internalization of GPCRs, but not other types of receptors, interacts constitutively with the Grks required for GPCR internalization, and inhibits the C-terminal phosphorylation of GPCRs carried out by these Grks, all independently of its catalytic Rac-GEF activity, and without obviously affecting Grk kinase activity. We propose that P-Rex1 inhibits GPCR trafficking by physically hindering the access of Grks to their target GPCRs.

## Discussion

Our study shows that P-Rex1 inhibits the agonist-stimulated internalization of GPCRs. This affects all GPCRs we tested, irrespective of the type of coupled heterotrimeric G protein. P-Rex1 inhibits the first step of GPCR trafficking, Grk-mediated receptor phosphorylation, implying subsequent steps are also abrogated, as β-arrestin cannot be recruited to non-phosphorylated GPCRs, and clathrin-mediated endocytosis depends on β-arrestin recruitment.^40^^,^^41^ P-Rex1 did not affect steady-state cell surface GPCR levels, nor total cellular GPCR levels, suggesting it plays no role in constitutive trafficking or degradation of GPCRs. P-Rex1 is also unlikely to affect GPCR recycling, as pilot experiments revealed normal localization of the recycling endosome marker Rab11 in HEK293-S1PR1 cells (not shown). Furthermore, the agonist-stimulated internalization of EGFR and PDGFR was normal, so P-Rex1 specifically limits GPCR trafficking.

P-Rex1 controls GPCR trafficking independently of its Rac-GEF activity. This is the first description of a GEF-activity independent role, although others were suggested previously. P-Rex1 binding to actin-remodeling protein FLII enhances FLII interaction with Rac1 to control cell/cell contacts, migration, and contraction.^53^ Although P-Rex1 binds FLII independently of its Rac-GEF activity, its Rac-GEF activity was required for downstream effects of FLII, so P-Rex1 functions as a Rac-GEF in that context. Similarly, *Prex1*^−/−^ mice have a melanoblast migration defect that affects skin pigmentation.^12^ The combined deletion of P-Rex1 and Rac1 caused a stronger phenotype, which suggested Rac1-GEF-independent roles,^74^ but melanoblast migration required the Rac-GEF activity,^12^ so P-Rex1 likely activates other Rac-GTPases, like RhoG, in this scenario. It is unsurprising that P-Rex1 has adaptor functions. Other Rac-GEFs also play important adaptor roles. Vav Rac-GEFs control NFAT-dependent transcription and integrin-mediated spreading in lymphocytes,^74^ and Tiam1 controls dendrite morphology in somatosensory neurons,^75^ independently of their Rac-GEF activities.

Our study is not the first to describe P-Rex1-dependent vesicle trafficking processes. P-Rex1 is required for the insulin-stimulated trafficking of glucose transporter 4 (GLUT4) in 3T3-L1 adipocytes, regulating glucose uptake. Dominant-negative Rac1 abolished this, suggesting that GLUT4 trafficking requires the P-Rex1 Rac-GEF activity.^76^ *Prex1*^−/−^ mouse platelets have defective dense granule secretion upon thromboxane stimulation,^77^ and P-Rex1 knockdown in endothelial cells impairs the epinephrine-stimulated secretion of Weibel-Palade bodies.^78^ Further research is needed to elucidate the underlying mechanisms in these processes.

We previously identified a direct interaction of P-Rex1 with the GPCR-adaptor protein Norbin,^79^^,^^80^ mediated through the PH domain and occurring *in vitro* and in cells. Norbin binding increases the P-Rex1 catalytic activity, and both proteins promote each other’s plasma-membrane localization.^80^ Norbin binds the C-terminal tails of many GPCRs, regulating GPCR signaling and trafficking.^79^ Norbin controls S1PR1 trafficking in PC12-S1PR1 cells,^51^ like P-Rex1. However, Norbin is not just a positive regulator of P-Rex1, and their roles in GPCR trafficking appear not to be linked. In mouse neutrophils, Norbin functions independently or even opposes P-Rex1, acting as a suppressor of neutrophil-mediated innate immunity, whereas P-Rex1 is required for immunity.^8^ Moreover, P-Rex1 only affects the agonist-induced internalization of GPCRs, whereas Norbin largely controls steady-state GPCR trafficking by limiting GPCR recycling, although it also modulates agonist-induced trafficking in some instances.^79^^,^^81^

P-Rex1 inhibits the phosphorylation of the C-terminal tail of GPCRs, which is required for receptor internalization. P-Rex1 binds Grk2, a major kinase responsible for GPCR phosphorylation, both in cells and *in vitro*, although it does not appear to regulate Grk2 activity. The P-Rex1 homolog P-Rex2 also inhibits GPCR trafficking and binds Grk2 in a manner independent of GEF activity. The less-pronounced effect of P-Rex2 on GPCR trafficking was likely caused by lower expression during transient transfections. S1PR1 is among GPCRs regulated by Grk2.^48^ To test whether Grk2 is the only Grk controlling S1PR1 trafficking in HEK293-S1PR1 cells, we compared the Grk2 inhibitor paroxetine with pan-Grk inhibitor CCG215022. Paroxetine had little effect at 10 μM, which was close to its half-maximal inhibitory concentration (IC_50_), whereas CCG215022 blocked S1PR1 trafficking. Higher concentrations of paroxetine were cytotoxic, limiting our interpretation of the relative importances of Grk2 and other Grks. The recently reported more potent Grk2 inhibitors^82^ could be used in the future. Even if multiple Grks control the trafficking of S1PR1, P-Rex1 is relevant as it not only interacts with Grk2 but also Grk5 and possibly other Grks.

P-Rex1 binding to Grk2 requires the DEP domains, which are central to P-Rex1 regulation and P-Rex1 downstream signaling.^3^^,^^4^ In addition to Grk2 binding, the DEP domains are involved in P-Rex1 binding to Gβγ and mTOR, and they harbor S436, a residue phosphorylated by PKA to prevent P-Rex1 activation. However, binding of the DEP domains to Grk2 was not sufficient for the regulation of GPCR trafficking; the PDZ and IP4P domains were also required, whereas the PH domain was dispensable. Others previously suggested that the isolated PDZ domains of P-Rex1 can bind S1PR1,^56^ we show that this is insufficient to affect receptor trafficking. P-Rex1 bound Grk2, but not the isolated catalytic domain of Grk2. Furthermore, P-Rex1 bound Grk5, which does not harbor a PH domain and cannot bind Gβγ. This suggested that P-Rex1 binds Grks at the conserved N terminus or RH domain. According to UniProt, the N termini of human Grk2 and Grk5 are more similar (67%) than the RH domains (46%) by primary sequence. However, 3D structures (RCSB PDB 3CIK and PDB 4NTB) showed that the RH domains are more highly conserved than suggested by the primary structure alone, so both N terminus and RH domain are the likely binding sites. More detailed mutagenesis or structural data will be required to identify the binding modus of P-Rex1 at Grks.

As P-Rex1 does not control Grk2 activity, we postulated that its direct interaction with Grk2 limits the agonist-induced internalization of GPCRs sterically, preventing access of Grk2 to the receptor. This was supported by P-Rex1 blocking Grk2 access to reconstituted S1PR1 in liposomes, preventing Grk2 from phosphorylating the receptor upon S1P stimulation. An alternative explanation was that Gβγ proteins, which bind both P-Rex1 and Grk2, are sequestered by P-Rex1, preventing them from activating Grk2. We tried gallein, a small-molecule Gβγ inhibitor,^83^ but saw no effects on the internalization of S1PR1-GFP, with or without P-Rex1 (not shown). We could not identify a suitable positive control to validate these experiments, so they did not allow us to conclude whether P-Rex1 interaction with Gβγ plays a role in regulating GPCR trafficking. An established construct of the Grk2 C terminus (bARK-CT)^84^ could be used instead in the future to block Gβγ function. However, several arguments speak against the need for Gβγ in P-Rex1 control of GPCR trafficking. Firstly, Gβγ proteins only outcompeted P-Rex1 binding to Grk2 at 4-fold molar excess, but not equimolar or lower stoichiometries. Secondly, P-Rex1 also binds Grk5, and unlike Grk2, Grk5 does not have a PH domain and cannot bind Gβγ.^68^ Thirdly, based on structural data, it was proposed that the P-Rex1/Gβγ binding mode differs from Grk2/Gβγ,^85^ so these Gβγ effectors may bind independently, although presumably not at the same time. Together, we therefore favor a mechanism whereby P-Rex1 binding to Grks blocks their access to GPCRs, preventing the kinases from phosphorylating the receptor. However, we cannot formally exclude a role for Gβγ. Unfortunately, testing this further by introducing point mutations in P-Rex1 to abolish Gβγ binding is not feasible. A cryo-EM structure of the C-terminal two-thirds of P-Rex1 in complex with Gβγ revealed that Gβγ makes extensive contacts over several domains, including DEP2, both PDZ, and the IP4P domain,^27^ and there is an additional Gβγ binding site in the DH-PH domain tandem.^29^ The mutation of large stretches in these domains would be required to abolish Gβγ binding.

As we propose a model of P-Rex proteins sequestering Grks away from GPCRs, the cellular stoichiometry of P-Rex and Grk family members is an important consideration. P-Rex1 protein is highly expressed in neutrophils (0.1% of cytoplasmic protein) and other leukocytes, neurons throughout the nervous system, and endothelial cells.^1^^,^^3^^,^^4^ P-Rex1 is also highly expressed in many cancers, including melanoma, glioblastoma, breast, prostate and colon cancer.^3^^,^^4^ P-Rex2 protein is highly expressed in the cerebellum and some endothelial cells, as well as in melanoma and breast cancer.^3^^,^^9^^,^^32^ Where P-Rex proteins are expressed, they often equal or outstrip the levels of Grks. For example, our quantitative proteomic analysis of mouse neutrophils showed that P-Rex1 is expressed at a similar level to Grk2 and Grk6, whereas P-Rex2 is absent, and other Grks are low.^86^ Quantitative proteomics of the human brain showed that P-Rex1 protein expression is higher than Grk2, followed by P-Rex2, Grk3, Grk5, and Grk6.^87^ Further proteomic analysis of various brain regions, lymph nodes, spleen, and lung also revealed comparable levels of P-Rex1 and P-Rex2 proteins to Grks.^88^^,^^89^ In some human cancers, P-Rex levels also outstrip Grks. P-Rex1 mRNA is higher than Grk mRNA in human glioblastoma and invasive breast cancer (www.proteinatlas.org/humanproteome/cancer), and there is similar expression of P-Rex1 to Grk proteins in MCF7 breast cancer and U-2 OS osteosarcoma cell lines.^90^ Therefore, in many normal and cancerous tissues and cell types where P-Rex1 and P-Rex2 are expressed, their levels would be sufficient to inhibit GPCR internalization based on stoichiometries alone.

Stoichiometry is not the only important factor when considering sequestration mechanisms. In the cell, local concentrations of P-Rex1 and Grks near the plasma membrane, particularly near the activated GPCR, are important and not a simple reflection of overall protein concentration. P-Rex1 is largely localized in the cytoplasm of basal cells and recruited to the plasma membrane by increased PIP_3_ and free Gβγ in the local environment of the activated receptor, resulting in local enrichment.^3^^,^^23^ We propose that this local enrichment of P-Rex1 in the 2D plane of the plasma membrane results in the out-competing of Grk binding to GPCRs. The absolute protein abundance in the relatively vast 3D volume of the cell is likely less important than the proteins in the vicinity of the active receptor. In addition to stoichiometries and subcellular enrichment, binding affinities will also play a role, as will interactions with additional binding partners, which are likely to exist but remain to be identified in this case. The binding affinity of P-Rex1 and Grk2 seems high, as it withstands stringent washing conditions, but the binding affinities of P-Rex1, Gβγ, and Grk complexes could be quantified in the future. Additional binding partners may further enhance the local concentration of P-Rex and Grk proteins near the GPCR. In addition, P-Rex1 will not need to affect every receptor in the cell. In a cancer setting, maintaining subsets of activated receptors at the plasma membrane would result in signal potentiation that increases invasiveness and subsequent metastasis, for example, in CXCR4-mediated cell motility, growth, and tumorigenesis in breast cancer cells.^14^ Therefore, the sequestration model proposed best represents the data we obtained and is physiologically relevant.

The synergistic mode of P-Rex1 activation by PIP_3_ and Gβγ makes it an ideal transducer of GPCR signals, with numerous examples in various biological systems. Together, PIP_3_ and Gβγ stimulate the Rac-GEF activity directly and cause translocation from the cytosol to the plasma membrane independently of the Rac-GEF activity.^1^^,^^3^^,^^23^ P-Rex1 is required for the fMLP- or C5a-stimulated activation of Rac2, ROS production, actin polymerization, chemokinesis, and chemotaxis in neutrophils,^1^^,^^2^^,^^5^^,^^91^ for C5a-and MCP1-stimulated Rac1 activity and chemotaxis in macrophages,^92^ thromboxane A2-, and thrombin-dependent secretion of dense granules in platelets,^77^ and SDF1-stimulated Rac1 activity and chemotaxis in endothelial cells.^93^ Furthermore, P-Rex1 is required for S1P-stimulated Rac1 and Akt activities, spreading, and neurite outgrowth in PC12-S1PR1 cells.^50^ Unfortunately, large parts of P-Rex1 are required to control GPCR trafficking, which hinders the design of mutants to abolish its trafficking role while retaining its signaling capacity. Therefore, it is difficult to dissect how much P-Rex1 mediates GPCR responses through signaling compared to trafficking, but P-Rex1 preventing Grk-dependent desensitization will result in retaining high levels of active GPCR at the plasma membrane and therefore prolong GPCR signaling. Thus, P-Rex1 plays a dual role in promoting GPCR responses.

These findings change our understanding of P-Rex biology. As a first example that they are functionally important on the organismal level, we recently found that P-Rex1 plays a GEF activity-independent role in glucose homeostasis at least in part by controlling the trafficking of the GPCR Gpr21 in the liver.^94^ Finally, our findings have implications for cancer therapy strategies. Many GPCRs, including S1PR1 and CXCR4, promote cancer growth and metastasis.^95^^,^^96^^,^^97^^,^^98^^,^^99^ Like most GEFs, P-Rex1 is difficult to target directly,^3^^,^^4^ whereas GPCRs are straightforward targets. Our findings predict that cancers with P-Rex1 overexpression will show high plasma membrane levels of GPCRs, and therapeutics against these GPCRs may already be in the clinic or in development.

### Limitations of the study

As large stretches of P-Rex1 bind Gβγ, we could not disrupt their interaction to investigate further the influence of Gβγ on P-Rex1 binding to Grks. Similarly, large parts of P-Rex1 are required to control GPCR trafficking, so we could not design mutants to dissect the relative importance of P-Rex1 in GPCR signaling compared to GPCR trafficking.

## Resource availability

### Lead contact

Further information and requests for resources and reagents should be directed to and will be fulfilled by the lead contact, Heidi C.E. Welch (heidi.welch@babraham.ac.uk).

### Materials availability

Newly generated materials associated with the paper are available from the lead contact.

### Data and code availability

• All data reported in this paper will be shared by the lead contact upon request.
• All targeted LS-MS data of P-Rex1 effects on S1PR1 phosphorylation at the C-terminal tail were deposited at Mendeley and are publicly available using https://doi.org/10.17632/tjncbccnxv.1.
• This paper does not report original code.
• Any additional information required to reanalyze the data reported in this work paper is available from the lead contact upon request.

## Acknowledgments

We thank Prof. Timothy Hla (Harvard University), Prof. Julie Pitcher (University College London), and Prof. Graham Ladds (University of Cambridge) for the constructs and helpful suggestions. We thank Dr. Simon Andrews from the Babraham Bioinformatics Facility, Dr. David Oxley from the Babraham Proteomics Facility, and staff of the Babraham Imaging Facility for their expert help. M.J.B. received a PhD studentship from the UK Biotechnology and Biological Sciences Research Council (BBSRC), E.H. a BBSRC iCASE PhD studentship in collaboration with Vernalis, P.I. a PhD studentship from the Cambridge Trust, E.M. a Summer Vacation Studentship from the British Society for Cell Biology, and E.T. a BBSRC iCASE PhD studentship in collaboration with AstraZeneca. The project was funded by the Institute Strategic Programme grant BB/P013384/1 from the 10.13039/501100000268BBSRC to the Babraham Institute Signalling Programme.

## Author contributions

M.J.B., E.H., P.I., R.P.M., E.A.M., K.H., and E.T. designed, performed, and analyzed the experiments. D.C.H, R.E.H., A.J.M., A.M., and H.C.E.W. planned and supervised the project and procured funding. M.J.B., E.H., and H.C.E.W. wrote the manuscript.

## Declaration of interests

The authors declare no competing interests.

## STAR★Methods

### Key resources table

**Table:** 

REAGENT or RESOURCE	SOURCE	IDENTIFIER
**Antibodies**		

CXCR4	Novus Biologicals	NB100-56437; RRID: AB_837929
EE	Babraham Bioscience Technologies	EE
EEA1	BD Biosciences	610456; RRID: AB_397829
EGFR	Abcam	ab52894; RRID: AB_869579
Flag	Sigma	F3165; RRID: AB_259529
Flag agarose-conjugated	Sigma	M8823; RRID: AB_2637089
GFP	Sigma	G6539; RRID: AB_259941
GFP	Abcam	ab290; RRID: AB_2313768
Grk2	Cell Signaling Technology	3982S; RRID: AB_330680
GST	Merck	Cytiva 27-4577-01; RRID: AB_771432
K-Ras	Sigma Aldrich	WH0003845M1; RRID: AB_1842235
Myc	Babraham Bioscience Technologies	myc
phospho-S324/S325 CXCR4	ECM Biosciences	CP435
phospho-S351/S353 S1PR1	7TM Antibodies	7TM0275A
P-Rex1	Marcus Thelen^1^	IRB, Bellinzona, Switzerland
P-Rex2	Welch lab^9^	78
Rab5	Abcam	ab18211; RRID: AB_470264
S1PR1	Abcam	ab11424; RRID: AB_298029
AF568 goat-anti-mouse IgG	Invitrogen	A-11031; RRID: AB_144696
HRP donkey anti-goat IgG	Santa Cruz	sc-3851; RRID: AB_641200
HRP goat anti-mouse IgG	Bio-Rad	1706516; RRID: AB_11125547
HRP goat anti-rabbit IgG	Bio-Rad	1706515; RRID: AB_11125142

**Biological samples**		

Tubulin purified from pig brain	Tetubio	T240

**Chemicals, peptides, and recombinant proteins**		

Aqua-Poly/Mount	Polysciences	18606–20
AY-NH2	Tocris	1487
CellMask Orange	Thermo Fisher Scientific	C10045
CCG215022	Seleckchem	CS-5870
ChromoTek agarose	Proteintech	bmab-20
Dimyristoyl phosphatidylcholine (DMPC)	Sigma	2663
Dulbecco’s Modified Eagle’s Medium	Gibco	41965–039
EGF	Sigma	11376454001
Escort IV	Sigma-Aldrich	L3287
G418 disulphate	Melford	G0175
Gallein	Tocris	3090
GLP-1	Tocris	5374
GST	Welch lab^80^	Babraham Institute
Hoechst 33342	Thermo Fisher Scientific	62249
JetPEI	Polyplus	101-10N
Magnetic high-capacity glutathione agarose	Merck	G0924
Octyl-b-D-glucopyranoside	Sigma	08001
OptiPrep solution	StemCell Technologies	07820
Paroxetine hydrochloride hemihydrate	Sigma	P9623
Pitstop2	Cayman Chemicals	23885
PDGF	Invitrogen	ABC125
Penicillin/streptomycin	Gibco	15140–122
Polyethylene glycol 8000	Thermo Scientific,	043443.36
Protein-A Sepharose	Sigma-Aldrich	P3391
Phosphate Buffered Saline (PBS)	Invitrogen	70011–036
ProLongGold Antifade	Life Technologies	P36934
Recombinant human wild type and mutant EE-P-Rex1	Welch lab^1^^,^^22^^,^^55^	Babraham Institute
Recombinant human GST-Grk2	Abcam	ab125620
Recombinant human GST-Grk5	Sigma	G8296
Recombinant prenylated EE-Gβ_1_γ_2_	Len Stephens^1^	Babraham Institute
Recombinant GST-S1PR1	Abcam	ab132072
S1P	Sigma	S9666
SDF1	Sigma	SRP3276
Sepharose	Sigma-Aldrich	4B-200
Soybean phosphatidylcholine (SBPC)	Sigma	P7443
X-tremeGENE 9	Roche	06366511001

**Critical commercial assays**		

ADP-Glo™ kinase assay	Promega	V6930
Clarity western ECL substrate	Bio-Rad	170–5060
Gateway Bac-to-Bac system	Invitrogen	11827–011
Site-directed mutagenesis kit	New England Biolabs	E0554

**Deposited data**		

Targeted LS-MS data of P-Rex1 effects on S1PR1 phosphorylation at the C-terminal tail	Mendeley	10.17632/tjncbccnxv.1

**Experimental models: Cell lines**		

Human Embryonic Kidney 293 (HEK293) cells	Stephens/Hawkins lab	Babraham Institute
Madin-Darby Canine Kidney (MDCK) cells with inducible expression of wild type or GEF-dead myc-P-Rex1	Angeliki Malliri^53^	CRUK Manchester Institute
PC12-S1PR1 cells, wild type or knock-out for *Prex1*	Welch lab^50^	Babraham Institute
Sf9 insect cells	Stephens/Hawkins lab	Babraham Institute

**Oligonucleotides**		

Primers (listed below)	Merck	N/A

**Recombinant DNA**		

pcDNA3-flag-Grk2	Julie Pitcher	University College London
pcDNA3-flag-Grk2-iCAT (aa 185–543)	Julie Pitcher	University College London
pcDNA3-S1PR1-GFP	Timothy Hla	Harvard University
pcDNA3.1-SNAP-GLP1R-mCherry	Graham Ladds	University of Cambridge
pcDNA3.1-PAR4-mCherry	Graham Ladds	University of Cambridge
pcDNA5FRT-EF-PDGFRβ-eGFP	Addgene	66790
pCMV3 EE-P-Rex1 and pCMV3 myc-P-Rex1 constructs	Welch lab^1^^,^^22^^,^^55^^,^^100^	Babraham Institute
pDEST10	Invitrogen	11806–015
pEGFP-N1-EGFR-GFP	Addgene	32751
pENTR3C	Thermo Fisher Scientific	A10464
pLSSmOrange-N1-hCXCR4-Orange	Addgene	110197

**Other**		

13 mm glass coverslips	Thermo Scientific	12392128
35 mm glass bottom dish	World Precision Instruments	FD35-100
16 × 102 mm ultra-clear ultracentrifuge tubes	Beckman Instruments	344661
Immobilon-P PVDF	Millipore	IPVH00010
LoBind tubes	Eppendorf	0030108132
Nunc T175 Easy flasks	Thermo Fisher Scientific	159920
0.5 mL Pierce 87777 detergent removal spin columns	Thermo Fisher Scientific	87777

### Experimental model and study participant details

Cell lines. Please see key resources table and method details sections.

### Method details

#### Expression vectors

Human P-Rex1 cDNA constructs with N-terminal myc or EE epitope tags in pCMV3 were described previously.^1^^,^^22^^,^^55^^,^^100^ mCherry-P-Rex1 was subcloned by replacing the EE-tag of pCMV3(EE)P-Rex1 with mCherry using Kpn1 and EcoR1. pCDNA3-S1PR1-GFP was a gift from Prof. Timothy Hla (Harvard University). pcDNA3.1-SNAP-GLP1R-mCherry and pcDNA3.1-PAR4-mCherry were gifts from Prof. Graham Ladds (University of Cambridge). pLSSmOrange-N1-hCXCR4-Orange (110197), pEGFP-N1-EGFR-GFP (32751) and pcDNA5FRT-EF-PDGFRβ-eGFP (66790) were from Addgene. Full-length pcDNA3-flag-Grk2 and pcDNA3 flag-Grk2-iCAT (aa 185–543) were gifts from Prof. Julie Pitcher (University College London). For the production of recombinant P-Rex2 proteins in Sf9 cells, human P-Rex2^31^ was subcloned into pENTR3C. Catalytically inactive (GEF-dead) P-Rex2^E30A,N212A^ was generated in pENTR3C using a site-directed mutagenesis kit (New England Biolabs, E0554) following the manufacturer’s instructions, with primers CGCGTGTGCGTGCTCAGCGCGCTCCAGAAGACCGAGCGG and GCTGTCTGTTCCAACATAGCCGAGGCCAAGAGACAGATG to introduce the E30A and N212A mutations, respectively. The wild type and GEF-dead P-Rex2 clones were recombined with pDEST10 (Invitrogen, 11806-015) to gain an N-terminal 6His tag and generate baculovirus using the Gateway Bac-to-Bac system (Invitrogen, 11827-011).

#### Western blotting

Proteins were transferred onto Immobilon-P PVDF (Millipore, IPVH00010) following SDS-PAGE. Primary antibodies were CXCR4 (Novus Biologicals, NB100-56437, 1:250), EE (clone Glu-Glu, Babraham Bioscience Technologies, 1:50), EEA1 (BD Biosciences 610456, 1:100), EGFR (Abcam, ab52894, 1:1000), flag (clone M2, Sigma, F3165, 1:6000), GFP (Sigma, G6539, 1:2000), Grk2 (Cell Signaling Technology, 3982S, 1:250), GST (Merck, Cytiva 27-4577-01, 1:1000), K-Ras (clone 3B10-2F2, Sigma Aldrich, WH0003845M1, 1:1000), myc (clone 9E10, Babraham Bioscience Technologies, 1:50), phospho-S324/S325 CXCR4 (ECM Biosciences, CP435, 1:250), phospho-S351/S353 S1PR1 (7TM Antibodies, 7TM0275A, 1: 500), P-Rex1^1^ (clone 6F12, from Prof. Marcus Thelen, IRB, Bellinzona, Switzerland, 1:50), P-Rex2^9^ (affinity-purified ‘78’, 1:10000), Rab5 (Abcam, ab18211, 1:1000), and S1PR1 (Abcam, Ab11424, 1:1000). Secondary antibodies were horseradish peroxidase (HRP)-coupled goat anti-rabbit (Bio-Rad, 1706515, 1:3000), goat anti-mouse (Bio-Rad, 1706516, 1:3000) or donkey anti-goat (Santa Cruz, sc-3851, 1:3000). Clarity Western ECL Substrate (Bio-Rad, 170–5060) was used. Where required, membranes were stripped in 25 mM glycine (pH 2.0), 1% SDS for 5 min at RT and reprobed. Coomassie staining (0.1% Coomassie brilliant blue R-250, 50% methanol, 10% acetic acid) of gels and membranes was used to control for protein loading. X-ray films were scanned, and band intensities were quantified by densitometry using Fiji (ImageJ).

#### Cell culture

Mammalian cell lines were used between 1 and 12 weeks in culture. Human Embryonic Kidney 293 (HEK293) cells were grown in Dulbecco’s Modified Eagle’s Medium (DMEM) (Gibco, 41965-039) supplemented with 10% fetal bovine serum (FBS), 100 U/mL penicillin and 100 μg/mL streptomycin (Gibco, 15140-122) at 37°C in a humidified incubator at 5% CO_2_. To generate HEK293 cells with stable expression of S1PR1-GFP (HEK293-S1PR1 cells), HEK293 cells were transfected with pcDNA.3-S1PR1-GFP using JetPEI and maintained in the same medium as HEK293 cells except with 500 μg/mL G418 disulphate (Melford, G0175) to select for resistance, and were FACS sorted to choose cells with moderate GFP signal. These cells were used for transient overexpression of P-Rex proteins, as they express low levels of endogenous P-Rex1 and P-Rex2.^33^^,^^81^^,^^100^ Madin-Darby Canine Kidney (MDCK) cells with doxycline (dox)-inducible expression of myc-tagged wild type or GEF-dead P-Rex1^53^ were grown in DMEM with 10% FBS, 100 U/mL penicillin, 100 μg/mL streptomycin, 1 μg/mL puromycin, and 500 μg/mL G418. Expression of wild type or GEF-dead P-Rex1 was induced by adding 1 μg/mL dox for 24 h. These cells were originally generated for the study of P-Rex1 in adhesion, polarity and migration,^53^ but were used here for transient overexpression of GPCRs, as all cells express either wild type or GEF-dead P-Rex1 upon dox treatment. PC12 (rat adrenal gland phaeochromocytoma) cells with stable expression of S1PR1-GFP (PC12-S1PR1 cells) which were either wild type or knock-out for *Prex1*,^50^ were grown in poly-D-lysine coated flasks, in DMEM with 10% horse serum, 5% FBS, 100 U/mL penicillin, 100 μg/mL streptomycin, 1× glutamine, and 500 μg/mL G418. Transient transfections were done using JetPEI (Polyplus, 101-10N) or X-tremeGENE 9 (Roche, 06366511001) following the manufacturers’ protocols. These cells were chosen, as all cells express S1PR1-GFP as well as expressing high levels of endogenous P-Rex1,^50^ so comparison of wild type and knockout cells allowed us to study the role of endogenous P-Rex1 in GPCR trafficking. Sf9 insect cells for the expression of recombinant proteins were cultured, lipofected using Escort IV transfection reagent (Sigma-Aldrich, L3287), baculovirus particles generated, amplified, and viral titers optimised for protein production as previously described.^55^

#### GPCR localization (imaging)

To measure S1PR1 internalisation in HEK293-S1PR1 cells which stably express S1PR1-GFP, the cells were seeded onto 13 mm coverslips (Thermo Scientific, 12392128) in 24-well plates (Nunc) and transiently transfected the following day with EE-tagged or myc-tagged P-Rex constructs using JetPEI, incubated for 21 h, washed, serum-starved for 6.5 h in DMEM, and then stimulated with 10 nM S1P for various periods of time. Cells were fixed in 4% paraformaldehyde (PFA) in 50 mM Pipes (pH 6.5), 1 mM EGTA, 10 mM MgCl_2_ for 15 min at RT, washed, permeabilised with 0.1% Triton X-100 in PBS for 10 min and washed again. Samples were blocked in PBS/0.5% BSA, incubated with EE antibody (clone Glu-Glu, Babraham Bioscience Technologies, UK, 1:10) or myc antibody (clone 9E10, Babraham Bioscience Technologies, UK, 1:10), washed again, and incubated with goat-anti-mouse AF568-IgG (Invitrogen, A-11031, 1:200), washed in PBS, rinsed in H_2_O, and mounted using ProLongGold Antifade (Life Technologies, P36934). Cells were imaged using the 60× objective of a Zeiss AxioImager D2 widefield microscope with AxioCam HRm camera. Duplicate coverslips were imaged for each condition, with focus on the apical cell surface, and 15 images acquired per coverslip. Images were blinded prior to analysis. To determine the localisation of S1PR1-GFP at the plasma membrane, images were either assessed semi-quantitatively by comparison to a panel of standard images, or were quantified using Volocity or CellProfiler software essentially as described,^51^ by generating a mask covering the entire cell and a second mask shrunk inwards by 0.619 μm (3 pixels), and calculating the GFP signal at the cell edge (mask 1 minus mask 2) as % of the total GFP signal (see also Figures S1 and S3).

In some experiments, HEK293-S1PR1 cells were preincubated for 30 min in the presence of 20 μM Pitstop2 (Cayman Chemicals, 23885), 10 μM paroxetine hydrochloride hemihydrate (Sigma, P9623), or 10 μM CCG215022 (Seleckchem, CS-5870), 10 or 50 μM Gallein (Tocris, 3090), or were mock-treated, prior to stimulation with 1 nM S1P or mock stimulation for 30 min, fixation in 4% methanol-free formaldehyde, and staining as here-above. Confocal images were acquired on Zeiss LSM880 or Olympus spinning disk microscopes.

To measure GPCR internalisation in MDCK cells with inducible expression of P-Rex1,^53^ cells were seeded onto 13 mm coverslips and transfected the next day using JetPEI to transiently express S1PR1-GFP, GLP1R-mCherry, PAR4-mCherry, or CXCR4-LSSmOrange. Alternatively, EGFR-eGFP or PDGFRβ-eGFP were expressed. The medium was changed 24 h after transfection, and 1 μg/mL dox was added 6 h later to half the samples to induce the expression of wild type or GEF-dead P-Rex1. 24 h after dox treatment, cells were serum-starved for 18 h in DMEM with 100 U/mL penicillin, 100 μg/mL streptomycin, 1 μg/mL puromycin, 500 μg/mL G418, and 0.1% fatty acid-free (FAF)-BSA. The cells were stimulated with the appropriate receptor agonists, namely S1P (Sigma, S9666) for S1PR1, GLP-1 (Tocris, 5374) for GLP1R, AY-NH2 (Tocris, 1487) for PAR4, SDF1 (Sigma, SRP3276) for CXCR4, EGF (Sigma, 11376454001) for EGFR, or PDGF (Invitrogen, ABC125) for PDGFβ, at various concentrations and periods of time, or were mock stimulated. The medium was aspirated, and cells were fixed in 4% PFA for 15 min, washed in PBS, stained with Hoechst 33342 (Thermo Fisher Scientific, 62249, 1:1000), washed again, mounted using ProLongGold Antifade and imaged using the 60× objective of a Nikon AR1 confocal microscope, with focus on a central plane. Receptor localisation was quantified as described here-above.

To measure S1PR1 internalisation in wild type and P-Rex1 deficient PC12-S1PR1 cells, cells were seeded onto 13 mm glass coverslips, serum-starved the next day for 14 h overnight in DMEM, 0.1% FAF-BSA, and then stimulated with 10 nM S1P for various periods of time, or mock stimulated, and fixed in 4% PFA. Samples washed in PBS, stained with Hoechst 33342, and mounted using Aqua-Poly/Mount (Polysciences, 18606-20). Cells were imaged using the 60× objective of a Nikon AR1 confocal microscope, with focus on a central plane. Duplicate coverslips were assessed per condition, and 5 images acquired per coverslip. Receptor localisation was quantified as described here-above.

#### GPCR localization (live-cell imaging)

HEK293-S1PR1 cells were seeded into 35 mm glass-bottom dishes (World Precision Instruments, FD35-100) and transfected the following day with mCherry-P-Rex1 using JetPEI. After 21 h, the cells were serum-starved in DMEM for 6 h. Cells were live-imaged using an Olympus CellR widefield imaging system at 37°C, 5% CO_2_, acquiring frames for GFP and mCherry every 30 s over 45 min. At the flash, an aspirator was used to gently replace the DMEM with DMEM containing 100 nM S1P, keeping a constant volume of 2 mL. Movies were processed using Fiji.

For cell membrane staining, HEK293-S1PR1 cells were seeded into 8-well chamber slides (ibidi, IB-80801). The following day, cells were serum-starved for 16 h overnight in DMEM, 0.1% FAF-BSA. Cells were then washed twice with PBS and incubated for 10 min with CellMask Orange (Thermo Fisher Scientific, C10045, 1:10,000) in DMEM. Cells were then washed twice with PBS and media replaced with phenol red-free DMEM with HEPES buffering. Live-cell imaging was performed on an Olympus spinning disk confocal microscope. One min after the initiation of imaging, S1P was added to a final concentration of 1 nM.

#### GPCR localization (cell fractionation)

Wild type and P-Rex1 deficient PC12-S1PR1 cells were seeded into poly-D-lysine coated T175 flasks, serum-starved the next day for 14 h overnight in DMEM, 0.1% FAF-BSA, and then stimulated with 5 nM S1P for 10 min, or mock-stimulated. The medium was aspirated, flasks were transferred onto metal trays on ice, rinsed with ice-cold PBS, and cells harvested by scraping into ice-cold PBS. Cells were centrifuged at 800 × g for 5 min at 4°C, resuspended in 3 mL of ice-cold detergent-free homogenisation buffer (25 mM sucrose, 20 mM Tricine-NaOH, 1 mM EDTA pH 7.8, 2 mM MgCl_2_, 2 mM DTT, 100 μM PMSF, and 10 μg/mL each of leupeptin, pepstatin-A, aprotinin and antipain) and homogenised in a Teflon-coated homogeniser by douncing. Samples were centrifuged at 800 × g for 10 min at 4°C. 20% OptiPrep solution (StemCell Technologies, 07820) in homogenisation buffer was added to the supernatant to give a concentration of 10% OptiPrep. Samples were loaded onto an OptiPrep step-gradient in 16 × 102 mm ultra-clear ultracentrifuge tubes (Beckman Instruments, Palo Alto, CA, 344661). The step gradient consisted of 5 layers of OptiPrep, 2.3 mL per layer, at 13.3%, 16.6%, 20%, 25% and 40% OptiPrep in homogenisation buffer from top to bottom. Samples were ultracentrifuged for 18 h at 90,000 × g at 4°C in a swinging bucket SW32.1Ti rotor with break/acceleration settings on minimum. 1 mL fractions were collected from each interphase. Proteins were precipitated from the fractions by addition of an equal volume of 25% trichloroacetic acid (TCA) and incubation for 30 min on ice. Samples were centrifuged at 13,225 × g for 15 min at 4°C, and the supernatant was removed. 1 mL of ice-cold acetone was added to each sample, and samples were centrifuged again. The supernatant was removed and the pellet left to air-dry for 20 min. Samples were resuspended in SDS-PAGE sample buffer, with addition of NaOH where necessary to adjust pH, and were analyzed by western blotting.

#### Targeted mass spectrometry of S1PR1 phosphorylation

HEK293-S1PR1 cells were seeded into Nunc T175 Easy flasks (Thermo Fisher Scientific, 159920). Half were transfected with pCMV3-EE-P-Rex1 and half with non-expressing control DNA using JetPEI. 21 h later, cells were serum-starved in DMEM for 6.5 h and then stimulated with 10 nM S1P for 10 min, or mock-stimulated. Flasks were transferred onto iced metal trays, washed in PBS, and cells were scraped into 1 mL ice-cold lysis buffer 1 (50 mM HEPES pH 7.2, 150 mM NaCl, 1% Triton X-100, 5 mM EDTA, 0.1 mM PMSF, 1 mM DTT, 20 mM β-glycerol phosphate, 25 mM NaF, 1 mM Na_3_VO_4_, 10 μg/mL each of leupeptin, aprotinin, pepstatin-A and antipain). Lysates were centrifuged at 110,000 × g for 30 min at 4°C, and the supernatant was incubated with 100 μL Sepharose beads (Sigma-Aldrich, 4B-200, prewashed in lysis buffer 1) for 20 min at 4°C with end-over-end rotation. The beads were sedimented at 18,000 × g for 30 s at 4°C, and the supernatant was transferred into precooled 1.5 mL Eppendorf tubes. 150 μL of supernatant was taken as a total lysate control. 6 μL GFP antibody (Abcam, ab290) was added to the remaining supernatant, and samples were incubated for 1.5 h at 4°C with end-over-end rotation before 60 μL of protein-A Sepharose (Sigma-Aldrich, P3391, prewashed in lysis buffer 1) was added, and samples were incubated for 1 h at 4°C with end-over-end rotation. The beads were sedimented at 18,000 × g for 30 s at 4°C, and the supernatant was removed. 150 μL of the supernatant was kept as a post-immunoprecipitation control. The beads were washed 5 times in lysis buffer 1, protein was eluted by 3 additions of 50 μL 0.1 M glycine, pH 2.5, and the pH was neutralised using 1 M Tris (pH 7.8 at 4°C). The eluates were centrifuged at 18,000 × g for 30 s at 4°C, and the supernatant was transferred to fresh precooled tubes. Boiling 4× SDS-PAGE buffer was added to final 1.3×, and samples were boiled for 10 min and snap-frozen in liquid nitrogen. Samples were subjected to tryptic digest, treated with titanium dioxide to enrich phosphopeptides, and analyzed by targeted liquid chromatography mass spectrometry (LC-MS). The ratio of phosphorylated to non-phosphorylated peptides was used to quantify the C-terminal phosphorylation of the GPCR.

#### Interaction of P-Rex1 with Grk2 in HEK293-S1PR1 cells

HEK293-S1PR1 cells were plated into T175 flasks, transfected with myc-P-Rex1 and/or flag-Grk2 or flag-Grk2-iCAT, the catalytic domain of Grk2 (aa 185–543),^67^ using jetPEI for 72 h, and then serum-starved in DMEM for 14 h. Cells were washed in PBS (Invitrogen, 70011-036), scraped, centrifuged at 10,000 × g for 30 s at 4°C, and resuspended in ice-cold lysis buffer 2 (50 mM HEPES, pH 7.2 at 4°C, 150 mM NaCl, 1% NP-40, 1 mM EDTA, 2 mM EGTA, 1 mM DTT, 0.1 mM PMSF, and 25 μg/mL each of leupeptin, pepstatin-A, aprotinin, and antipain). The lysate was incubated on ice for 10 min with intermittent vortexing, cleared by centrifugation at 10,000 × g for 3 min at 4°C, and the supernatant recovered. For a total lysate control, boiling 4× SDS-sample buffer was added to 75 μL of cleared lysate, samples boiled for 5 min, and frozen in liquid nitrogen. The rest of the lysate was transferred into precleared 2 mL LoBind tubes (Eppendorf, 0030108132) and precleared with 10 μg prewashed magnetic ChromoTek agarose (Proteintech, bmab-20) for 30 min at 4°C with end-over-end rotation. The beads were removed magnetically, and the supernatant was transferred into fresh tubes and incubated with 10 μg prewashed magnetic anti-flag agarose beads (Sigma, M8823) for 60 min at 4°C with end-over-end rotation. 75 μL of the supernatant was retained as a ‘supernatant’ control and processed like the total lysate control. The beads were washed 4 times and resuspended in 30 μL boiling 1.3× SDS-sample buffer, boiled for 5 min, and frozen in liquid nitrogen. Samples were analyzed by western blotting with P-Rex1 and flag antibodies.

Co-immunoprecipitation of P-Rex1 with S1PR1-GFP was performed the same way, except that HEK293-S1PR1 cells were transfected with myc-P-Rex1 alone, or mock transfected, serum starved, and stimulated with 100 nM S1P for 10 min, or mock-stimulated. S1PR1-GFP was immunoprecipitated using magnetic GFP-trap agarose, and samples were analyzed by western blotting with myc and S1PR1 antibodies.

#### Recombinant proteins

Recombinant human wild type and mutant EE-P-Rex1 proteins, purified from baculovirus-infected Sf9 cells using their EE tag, were as previously described.^1^^,^^22^^,^^55^ Sf9-cell-derived purified recombinant prenylated EE-Gβ_1_γ_2_ proteins were a kind gift from Len Stephens (Babraham Institute) and were as previously described.^1^ Recombinant human wild type and GEF-dead His-P-Rex2 proteins were purified from baculovirus-infected Sf9 cells using their His tag. Pellets from 400 mL Sf9 cell cultures infected with high titer baculovirus (see above) were thawed into 25 mL ice-cold lysis buffer 3 (PBS, 1% Triton X-100, 25 mM NaF, 20 mM β-glycerophosphate, 1 mM DTT, 0.1 mM PMSF and 10 μg/mL each of antipain, pepstatin A, leupeptin, aprotinin), lysed on ice for 5 min, and ultracentrifuged at 200,000 × g for 1 h at 4°C. The supernatant was incubated with prewashed Ni-NTA agarose for 90 min at 4°C and with end-over-end rotation. Beads were washed 3 times in ice-cold 2× PBS, 1% Triton X-100 and 4 times in wash/elution buffer (PBS, 10% glycerol, 1 mM DTT, 0.01% azide, 20 mM imidazole). For elution, wash/elution buffer containing 600 mM imidazole was added, and samples were incubated for 10 min on ice. Samples were centrifuged at 800 × g for 1 min at 4°C and the supernatant recovered. A second elution was performed with 300 mM imidazole and pooled with the first. To remove the imidazole, a PD-10 desalting column (GE Healthcare Life Sciences, 52130800) was used according to the manufacturer’s instructions. Desalted protein was concentrated using a 100 kDa Amicon Ultra filter (Merck, UCF210024). Once the sample volume was reduced to 100 μL, 2 mL of equilibration buffer (1× PBS, 10% glycerol, 1 mM DTT, 1 mM EGTA, 0.01% azide) were added, and the samples concentrated again. Glycerol was added to 50%, and protein for GEF activity assays was supplemented with 2 mg/mL FAF-BSA. To test the quality of the purified P-Rex2 proteins, Rac-GEF activity was measured using a liposome-based assay as previously described.^1^^,^^31^^,^^55^ GST was purified from *E. coli* as previously described.^80^ Sf9 cell-derived recombinant human GST-Grk2 was from Abcam (Abcam, ab125620).

#### Direct binding of P-Rex proteins to Grk2 and Grk5

5 pmol P-Rex1 or P-Rex2 protein were incubated with 5 pmol GST, GST-Grk2, or GST-Grk5 in a volume of 20 μL in detergent-free buffer (50 mM HEPES, pH 7.2 at 4°C, 150 mM NaCl, 1 mM EDTA, 2 mM EGTA, 1 mM DTT, 0.1 mM PMSF, and 25 μg/mL each of leupeptin, pepstatin-A, aprotinin, and antipain) for 1 h on ice, with frequent vortexing. For experiments with P-Rex1 mutants, protein amounts were reduced to 2.5 pmol per sample. A 2 μL aliquot was taken as the ‘reaction mix’ control. Boiling 1.3× SDS-sample buffer was added, and the sample was boiled for 5 min and frozen in liquid nitrogen. The remaining reaction mix was added to 300 μL detergent-free buffer in LoBind tubes containing 5 μL prewashed magnetic high-capacity glutathione agarose (Merck, G0924) and incubated for 45 min at 4°C with end-over-end rotation. The beads were sedimented using a magnet, and 75 μL of the supernatant was retained as a ‘supernatant’ control, which was processed like the ‘reaction mix’ control. The beads were washed four times in lysis buffer 2, boiling 1.3× SDS-sample buffer was added, and samples were boiled for 5 min, and frozen in liquid nitrogen. Samples were analyzed by western blotting using P-Rex1 or P-Rex2 and GST antibodies.

In experiments testing the effects of Gβγ proteins on P-Rex1 binding to Grk2, increasing concentrations of Sf9-cell-derived purified recombinant prenylated EE-Gβ_1_γ_2_ proteins, up to 20 pmol per sample, were added during the binding assay. The final assay concentration of cholate from the Gβγ storage buffer was 0.008% and was kept constant between all samples. The EE-Gβ_1_γ_2_ proteins were detected by western blotting with EE antibody.

#### Grk2 kinase activity

To measure the catalytic activity of Grk2, the ADP-Glo kinase assay kit (Promega, V6930) was used with tubulin as the substrate. 40 nM human recombinant GST-Grk2 and/or 40 nM EE-P-Rex1 proteins were incubated with 150 nM tubulin purified from pig brain (Tetubio, T240) and 400 μM ATP in kinase buffer (40 mM Tris, pH 7.5 (RT), 20 mM MgCl_2_, and 0.1% BSA), in a volume of 25 μL for 30 min at 30°C. Controls included samples without protein and with kinase detection reagent only. To control for potential effects of the storage buffers of EE-P-Rex1 (PBS, 1 mM EGTA, 1 mM DTT, 50% glycerol, 0.01% sodium azide) and GST-Grk2 (0.79% Tris HCl, 0.88% NaCl, 0.31% glutathione, 0.002% PMSF, 0.004% DTT, 0.003% EDTA, 25% glycerol), the buffers were added to samples without protein at the equivalent dilution. After the incubation, 25 μL ADP-Glo reagent was added for 40 min at RT to deplete any remaining ATP. 50 μL of kinase detection reagent was added for a further 40 min, and luminescence was measured in a PHERAstar FS luminometer (BMG Labtech).

#### Reconstitution of S1PR1 into liposomes

Protocols for the reconstitution of functional GPCRs into liposomes were adapted from^69^^,^^70^^,^^71^^,^^72^ for use with S1PR1. Liposomes were prepared by air-drying a mixture of 24 μL soybean phosphatidylcholine (SBPC) (Sigma, P7443) and 10 μL dimyristoyl phosphatidylcholine (DMPC) (Sigma, 2663) from 10 mg/mL stocks in CHCl_3_, followed by the addition of 50 μL 10 mM Tris, pH 7.5, and bath sonication to clarity to yield liposomes comprised of 7 mM SBPC and 3 mM DMPC. 3 μg purified recombinant GST-S1PR1 (Abcam, ab132072, 130 pmol in 100 μL 65 mM Tris pH 8) were added to 30 μL of the liposomes, together with 100 mM NaCl, 2 mg/mL FAF-BSA, 0.85% octyl-b-D-glucopyranoside (Sigma, 08001), 0.1 mM PMSF, and 0.25 mM DTT, in a final volume of 150 μL, giving 1.4 mM SBPC and 0.6 mM DMPC. 2 μL were taken as the ‘input control’, made up to 40 μL with boiling 1.3× sample buffer, boiled, snap-frozen in liquid N_2_, and stored at −80°C. The remaining sample was split in two, and 3 μL of 7.5 μM S1P (in 10 mM Tris pH 7.5) was added to one to give 300 nM S1P. From this point on, S1P was added to ‘+S1P’ samples at each subsequent step of the reconstitution procedure to maintain 300 nM. Samples were incubated on ice for 30 min. During the incubation, 0.5 mL Pierce 87777 detergent removal spin columns (ThermoFisher Scientific, 8777) were washed once with 10 mM Tris pH 7.5, 100 mM NaCl, 2 mg/mL FAF-BSA and then incubated in the same buffer for 10 min at RT to block the columns. The columns were equilibrated once in same buffer except without BSA, the samples were applied, and the flow-through was collected according to the manufacturer’s instructions. 2 μL ‘column controls’ were taken and treated like the ‘input control’ above. 75 μL 40% polyethylene glycol 8000 (Thermo Scientific, 043443.36, w/v in 10 mM Tris pH 7.5) were added to each sample to aid vesicle fusion and increase the recovery of the GPCR in liposomes,^70^ and samples were incubated for 10 min at RT with occasional vortexing. Samples were diluted to 1.5 mL in ice-cold 10 mM Tris pH 7.4, 100 mM NaCl, 0.1 mM PMSF, 0.25 mM DTT, and ultracentrifuged at 250,000 × g in a Beckman Ultima benchtop ultracentrifuge for 2 h at 4°C. Pellets were resuspended in 80 μL kinase assay buffer (20 mM Tris-HCl pH 7.5 RT, 5 mM MgCl_2_, 2 mM EDTA)^71^ supplemented with 0.1 mM PMSF, 0.25 mM DTT, 10 mM β-glycerophosphate, and 10 μg/mL each of antipain, aprotinin, leupeptin, and pepstatin (KAB^+^). 2 μL ‘pellet controls’ were taken and treated like the ‘input control’ above.

To test for effects of P-Rex1 on Grk2 accessing the liposomal S1PR1, 1.4 pmol recombinant GST-Grk2 were preincubated with or without 2.5 pmol recombinant EE-P-Rex1 in KAB^+^ for 10 min at 30°C in a volume of 4 μL per sample. 20 μL of the reconstituted liposomal S1PR1 and 1.2 μL 2.5 mM ATP (in 25 mM Tris-HCl pH 7.5) were added, as well as 1.2 μL 7.5 μM S1P for ‘+S1P’ samples, and KAB^+^ was added to a final volume of 30 μL per sample. Final assay concentrations were ∼170 nM liposomal GST-S1PR1, 100 nM EE-P-Rex1, 47 nM GST-Grk2, 100 μM ATP, and 300 nM S1P. Samples were incubated for 30 min 30°C. 10 μL boiling 4× sample buffer was added, and samples were boiled, snap-frozen, and stored at −80°C. Samples were analyzed by western blotting with antibodies to phospho-S351/S353 of S1PR1 (7TM Antibodies, 7TM0275A, 1: 500), GST antibodies to detect GST-Grk2 and total GST-S1PR1, and with P-Rex1 antibodies.

### Quantification and statistical analysis

Data were tested for normality of distribution to determine if parametric or non-parametric methods of analysis were appropriate. For comparison of two groups, paired Student’s *t* test was used, whereas for comparison of multiple groups, one-way or two-way ANOVA was used, as appropriate, with repeated measures followed by post-hoc test with multiple comparisons correction. Parameters with values of *p* ≤ 0.05 were considered to differ significantly. In the figures, ^∗^ indicates *p* < 0.05, ^∗∗^*p* < 0.01, ^∗∗∗^*p* < 0.001, and ^∗∗∗∗^*p* < 0.0001. Results are presented as mean ± standard error of the mean (SEM). The number of experimental repeats is indicated in the figure legends. Statistical analysis and plotting of graphs were performed in GraphPad Prism 10.

## Supplemental information

Document S1. Figures S1–S10

Document S2. Article plus supplemental information
