# Supplementary material for: P-Rex1 limits the agonist-induced internalization of GPCRs independently of its Rac-GEF activity
Source: Cell Rep. 2025 Oct 14;44(10):116403. doi: 10.1016/j.celrep.2025.116403 (PMC12569377; doi:10.1016/j.celrep.2025.116403)
Supplement: Document S1. Figures S1–S10 [file mmc1.pdf]

Cell Reports, Volume *44*

Supplemental information

P-Rex1 limits the agonist-induced internalization

of GPCRs independently of its Rac-GEF activity

Martin J. Baker, Elizabeth Hampson, Priota Islam, Ruben Pelaez Moral, Eve A. Maunders, Kirsti Hornigold, Elpida Tsonou, Angeliki Malliri, David C. Hornigold, Roderick E. Hubbard, Andrew J. Massey, and Heidi C.E. Welch

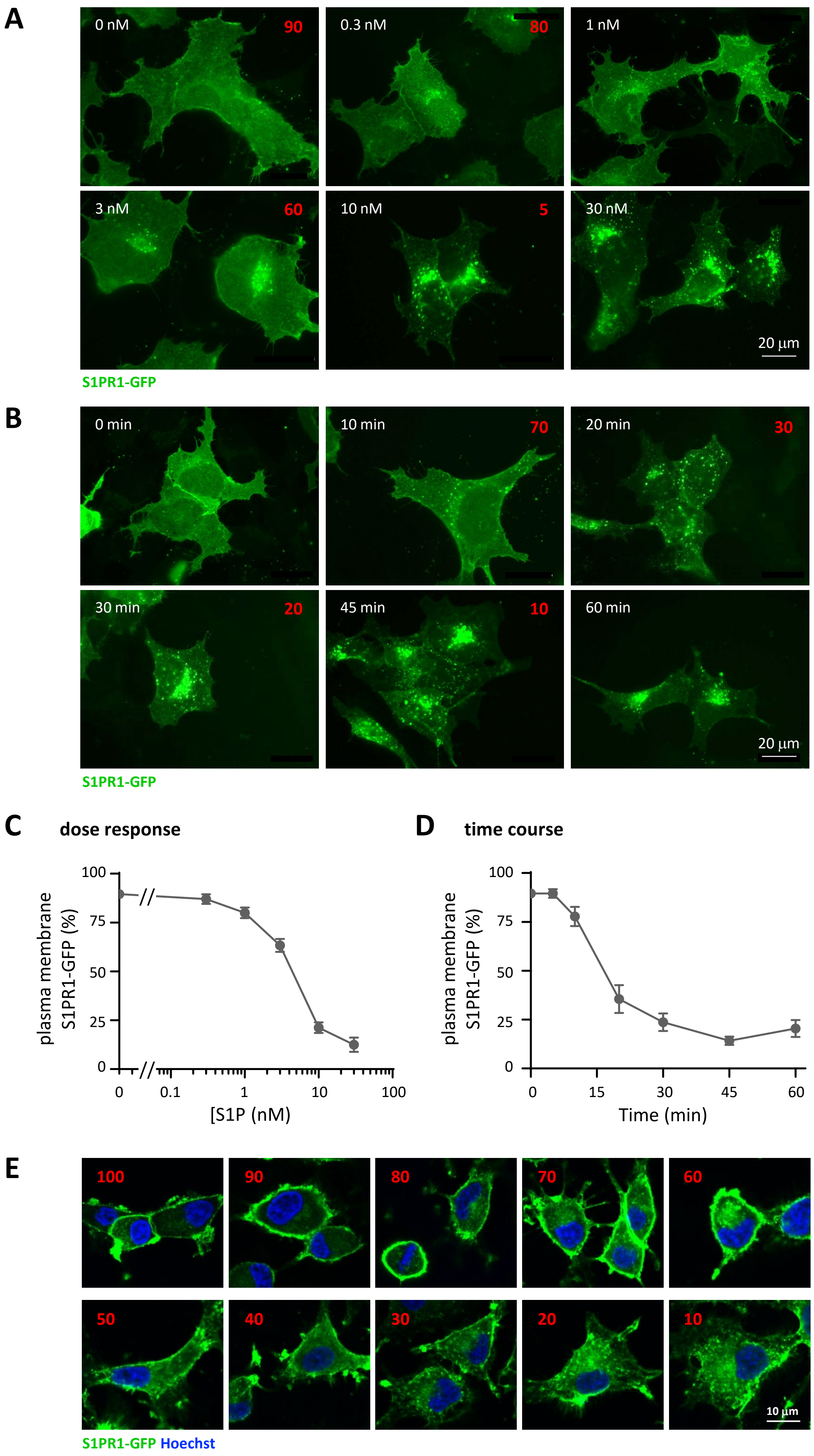

Supplemental Figure 1

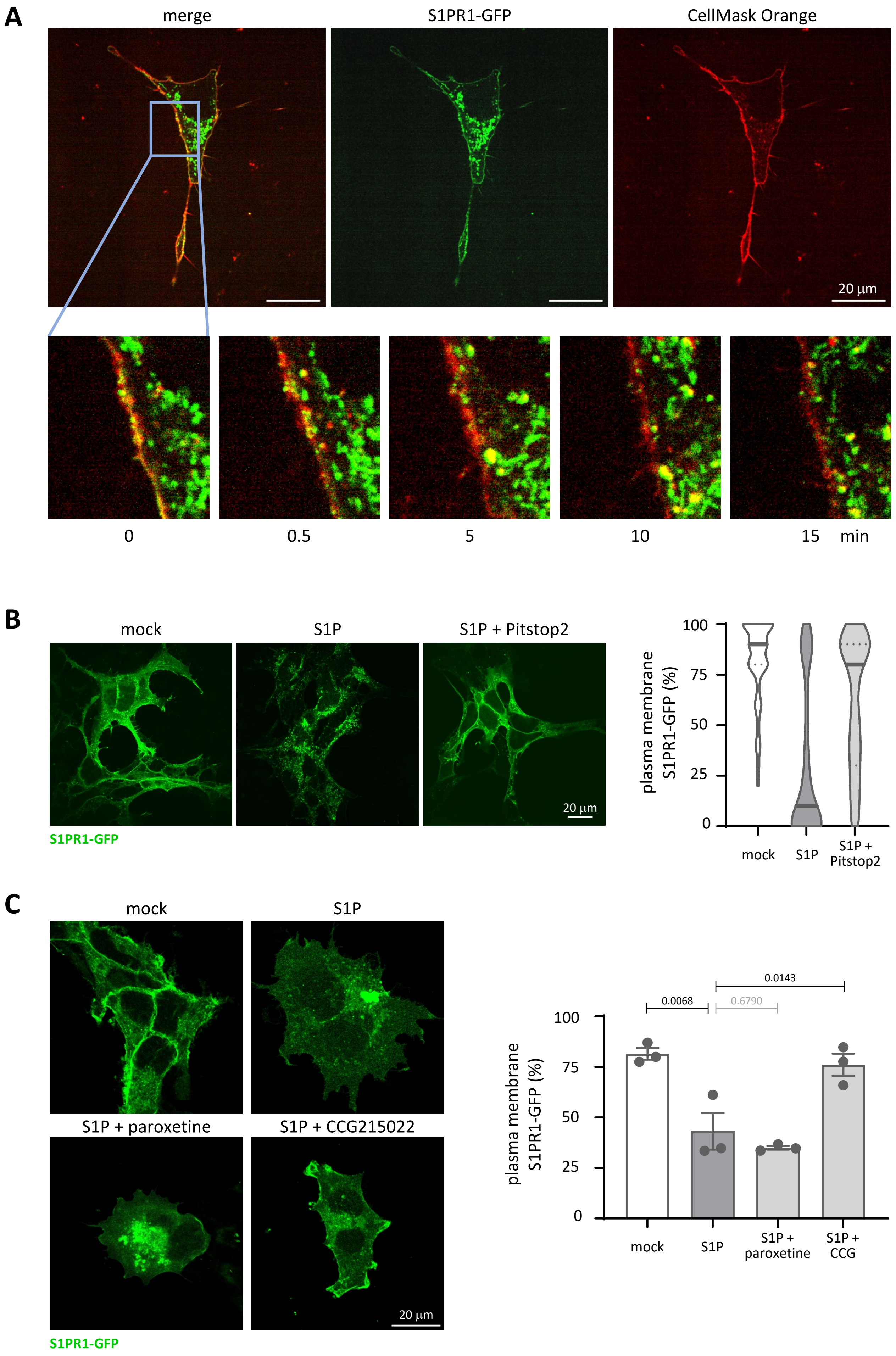

Supplemental Figure 2

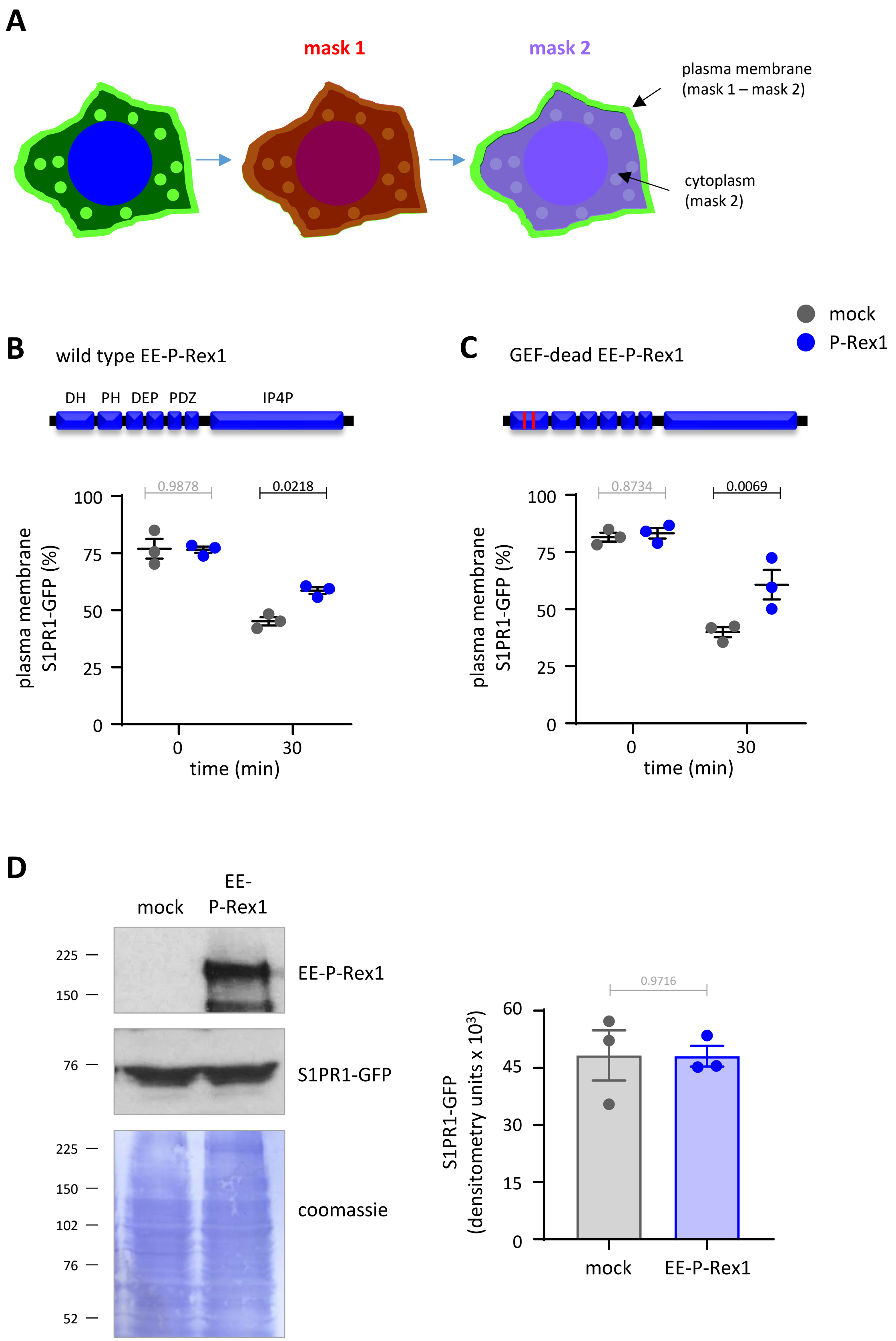

Supplemental Figure 3

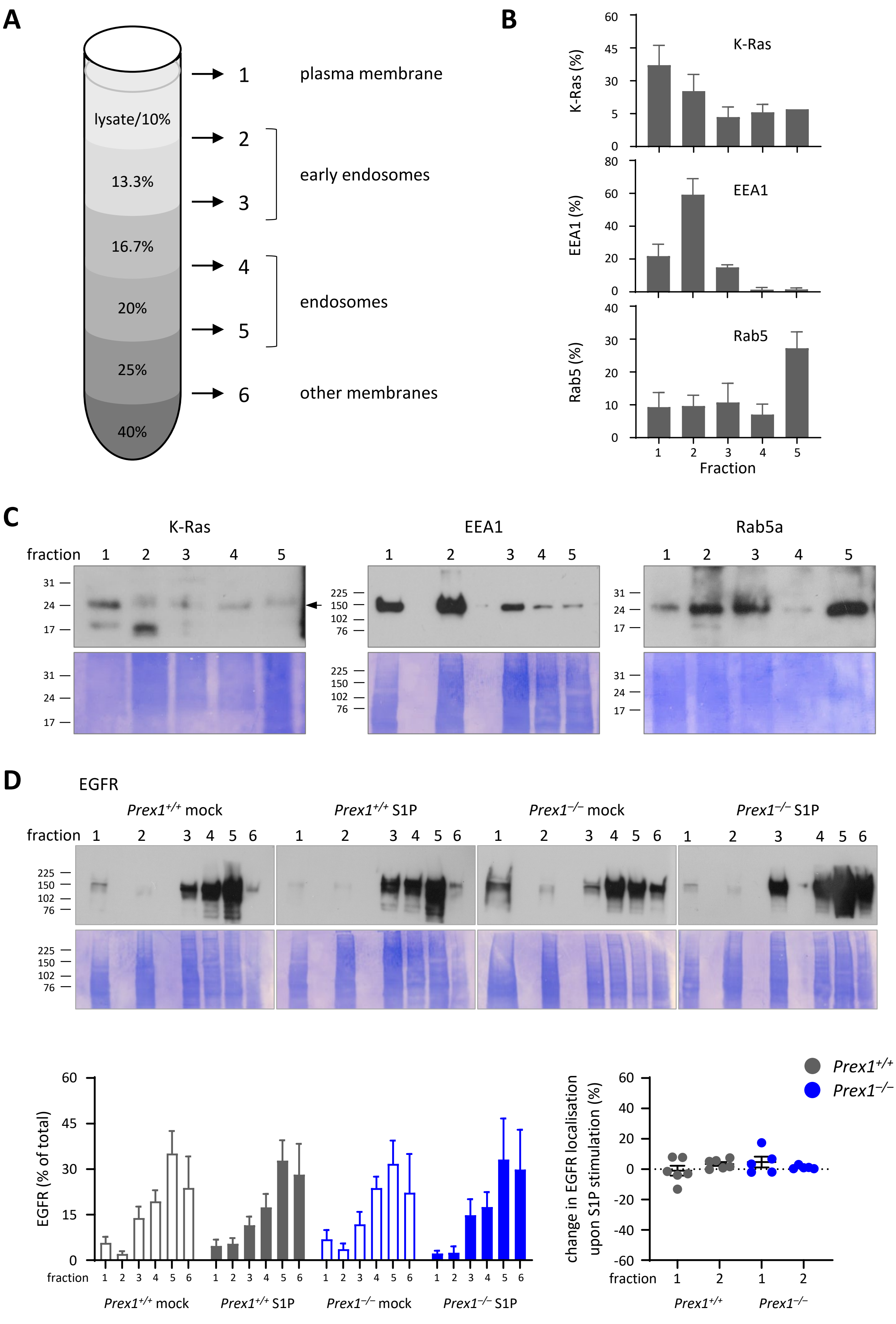

Supplemental Figure 4

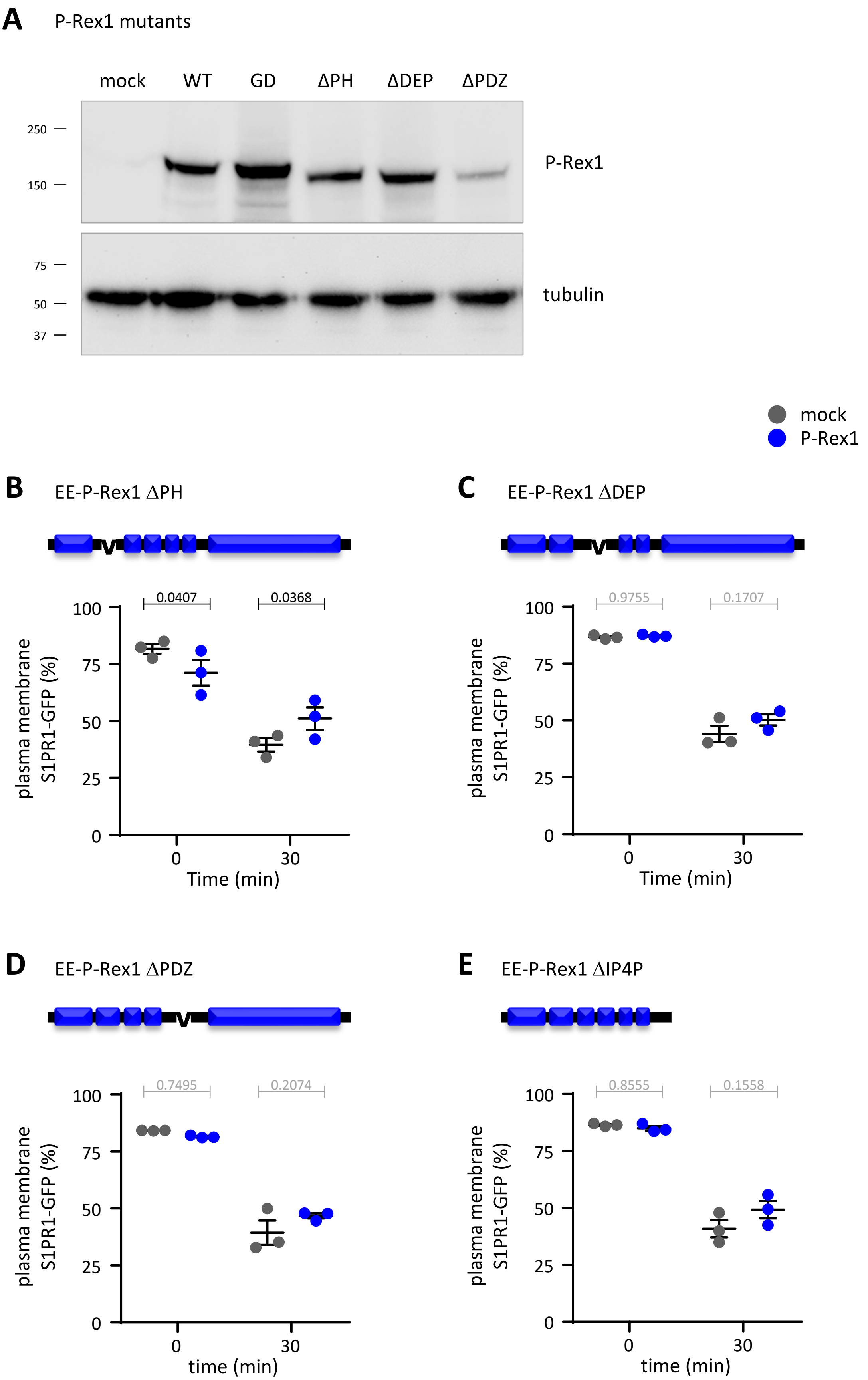

Supplemental Figure 5

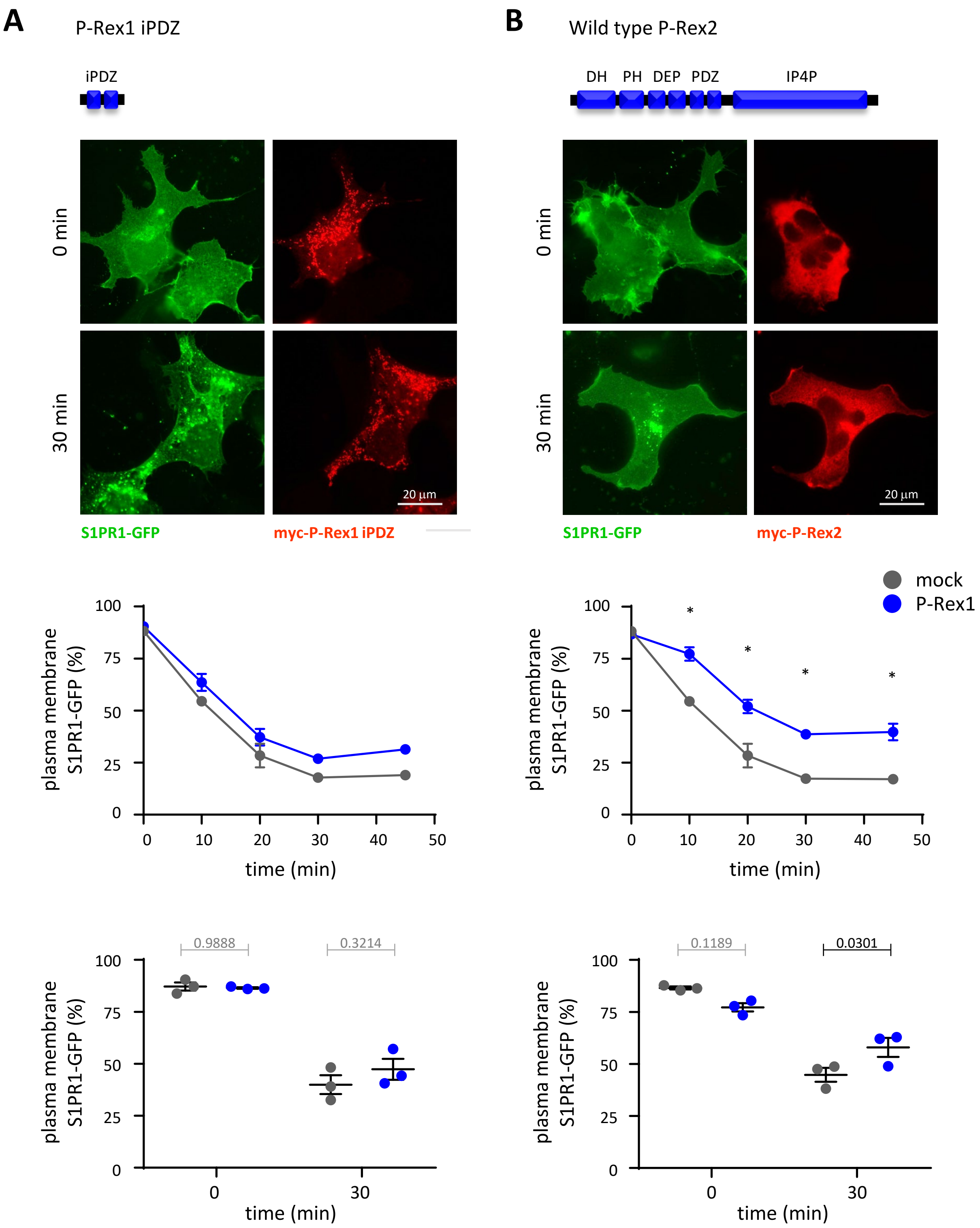

Supplemental Figure 6

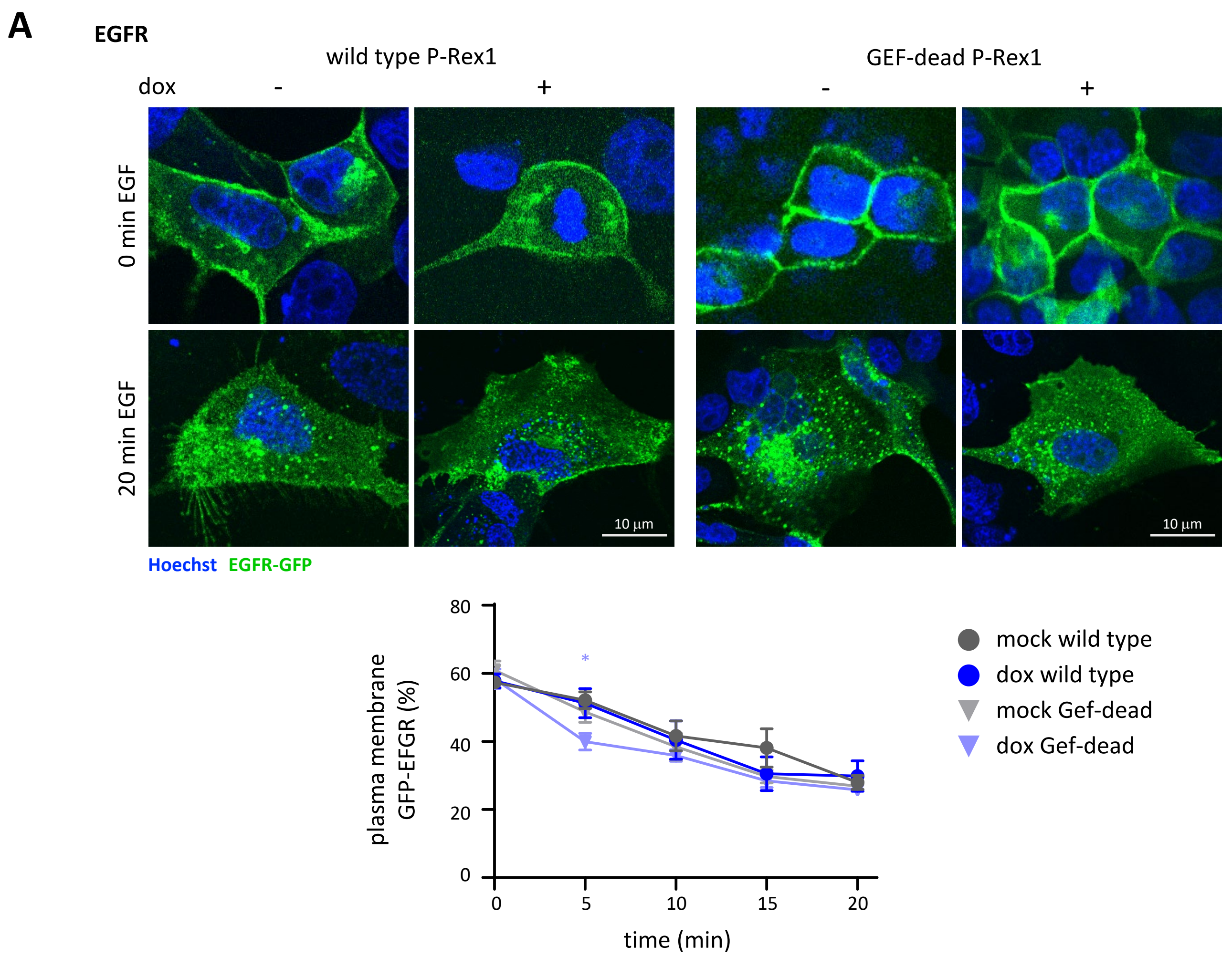

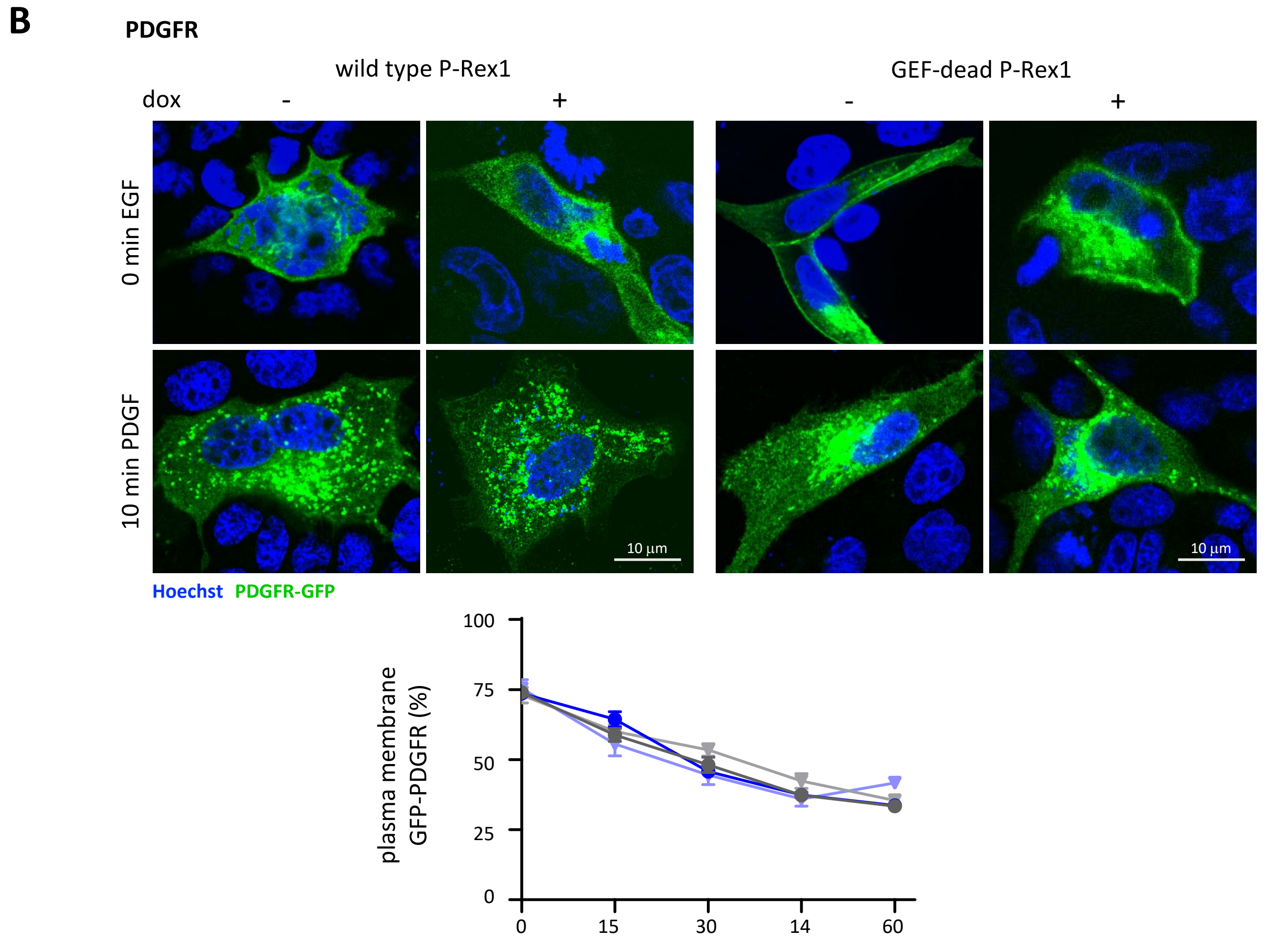

Supplemental Figure 7

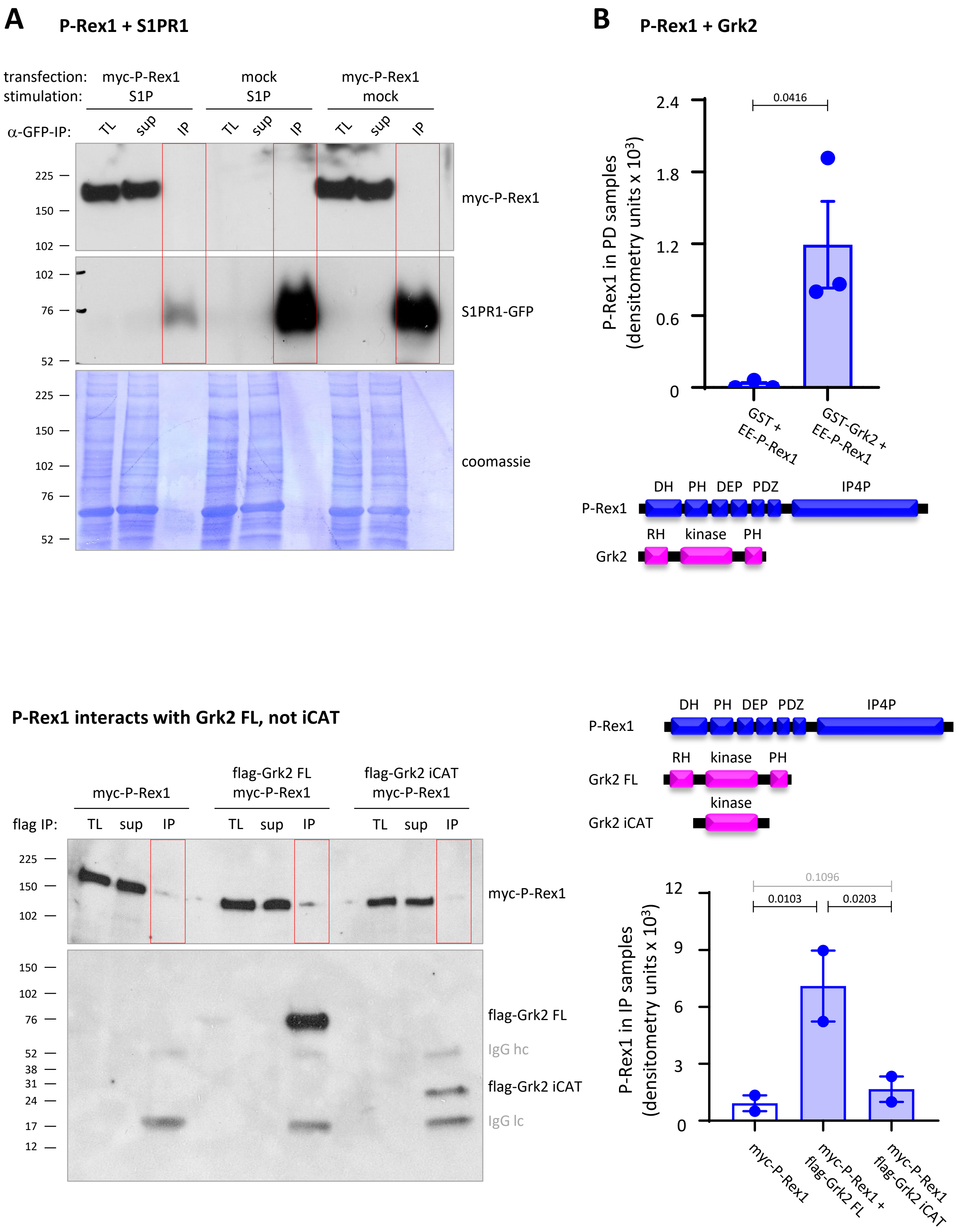

Supplemental Figure 8

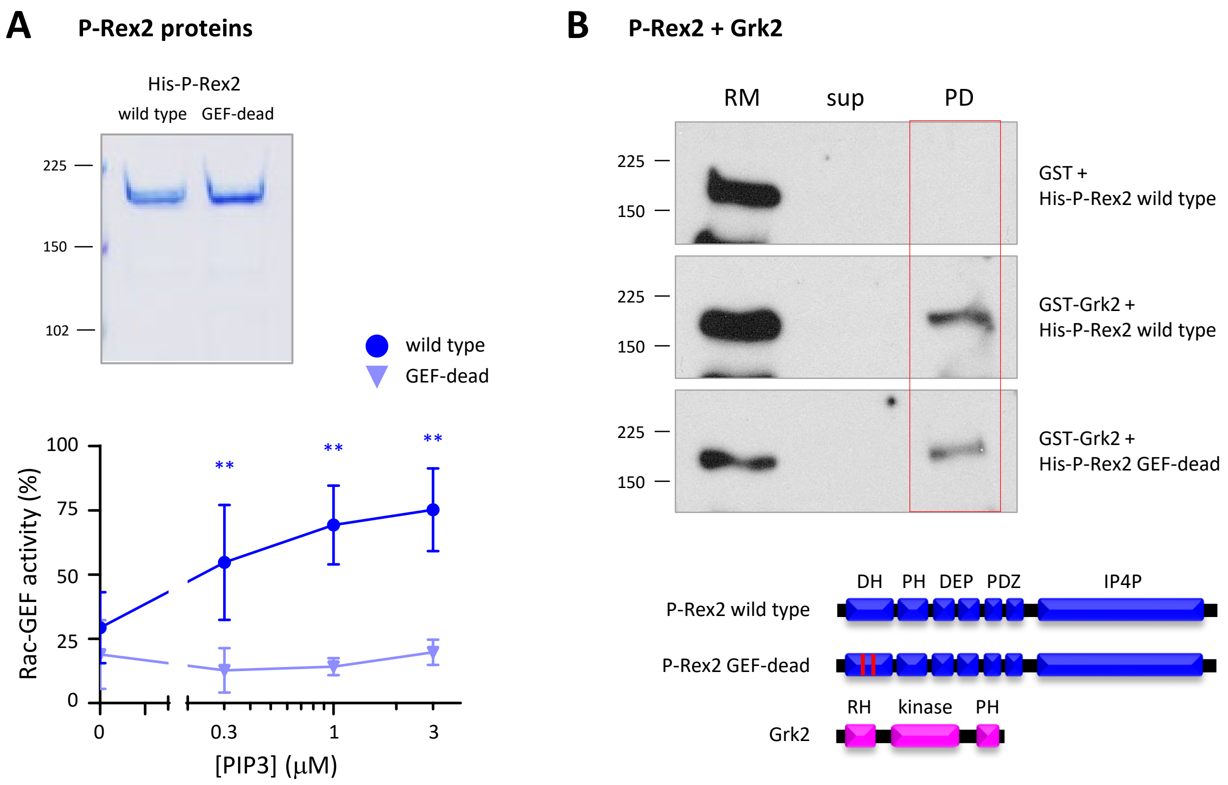

Supplemental Figure 9

Gβγ competition

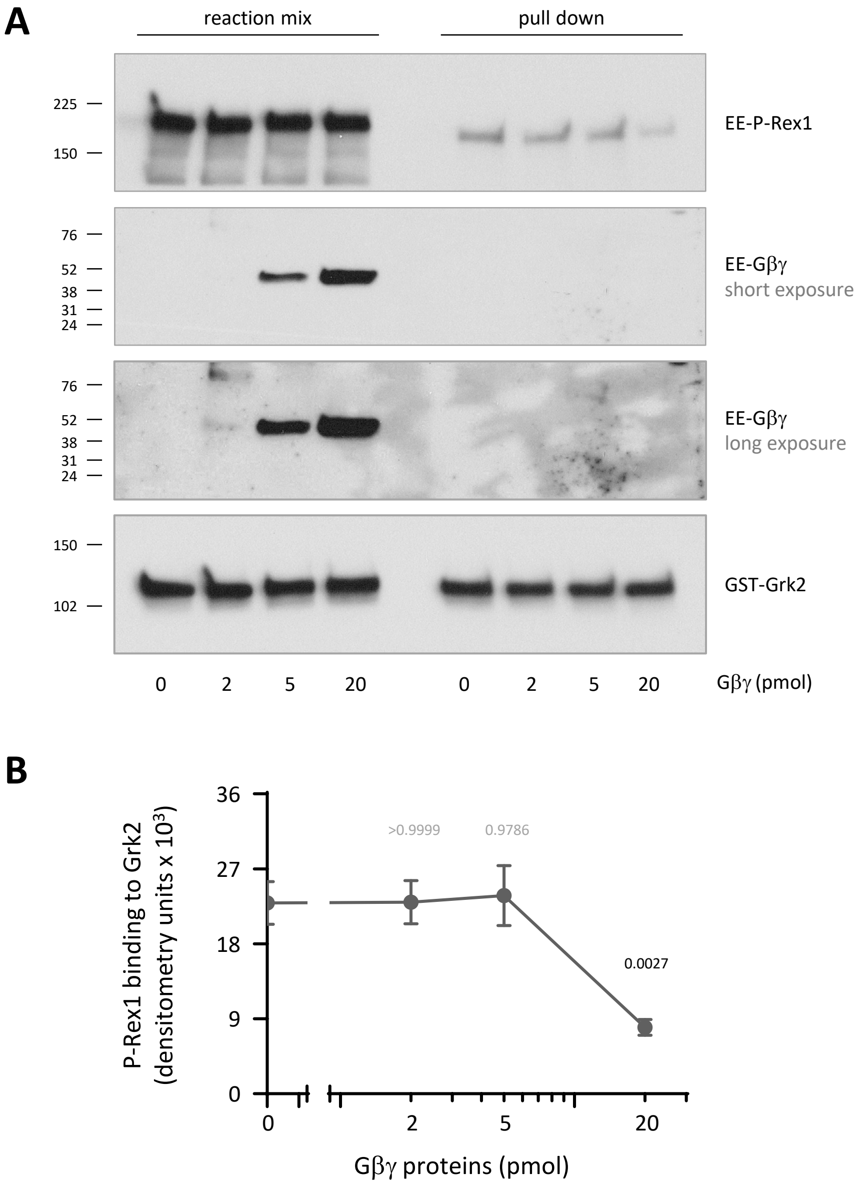

Supplemental Figure 10

Supplemental Figure Legends

Supplemental Figure 1. S1PR1-GFP is internalised in a dose- and time-dependent manner upon S1P stimulation of HEK293-S1PR1 and PC12-S1PR1 cells. HEK293-S1PR1 cells were serum-starved for 6.5 h and stimulated with the indicated concentrations of S1P for 30 min **(A, C)** or stimulated with 10 nM S1P for the indicated periods of time **(B, D)**, fixed and imaged by widefield fluorescence microscopy with focus on the apical cell surface. (A,B) show representative images, which show the sheet-like appearance of S1PR1-GFP typical for plasma membrane proteins imaged by widefield microscopy and the vesicular localisation in S1P-stimulated cells. Images with red numbers (in %) form part of a panel of standard images used to determine how much receptor is localised at the plasma membrane. (C,D) Quantification of S1PR1-GFP at the plasma membrane by comparison of blinded images to a panel of standard images. Data are mean ± SEM of cells from one of two similar pilot experiments. **(E)** Representative images for the analysis of S1PR1-GFP localisation by confocal microscopy. PC12-S1PR1 were serum-starved for 14 h and stimulated for 10 min with S1P concentrations ranging from 0-50 nM, fixed, stained with Hoechst 33342, and imaged by confocal fluorescence microscopy with focus on a central plane. Images show the S1PR1-GFP ring at the cell periphery typical for plasma membrane proteins imaged by confocal microscopy, and the vesicular localisation in S1P-stimulated cells. Red numbers (in %) denote the amount of S1PR1-GFP at the plasma membrane.

Supplemental Figure 2. The S1P-dependent internalisation of S1PR1 by clathrin-mediated endocytosis requires Grk activity. HEK293-S1PR1 cells were serum-starved for 16 h**,** stimulated with 1 nM S1P for 30 min, and imaged by confocal fluorescence microscopy with focus on a central plane, either by live-imaging (A) or after fixing (B,C). **(A)** Stills from Supplemental Movie 1 of cells incubated with CellMask Orange to label the plasma membrane prior to live-imaging of S1PR1-GFP localisation. Magnifications of the area indicated by the blue box are shown over time. **(B)** Pitstop2. Cells were preincubated in the presence or absence of 20 µM Pitstop2, an inhibitor or clathrin-mediated endocytosis, for 30 min prior to stimulation with 1 nM S1P, or mock-stimulation, as indicated. Representative images are shown. Images from one experiment were quantified for the plasma membrane localisation of S1PR1-GFP as described in Supplemental Figure 1E. **(C)** Grk activity dependence. Cells were preincubated in the presence or absence of 10 µM of the Grk2 inhibitor paroxetine or pan-Grk inhibitor CCG215022 for 30 min, or were mock-treated with DMSO, prior to stimulation with 1 nM S1P or mock stimulation, as indicated. Representative sum z-projection images are shown. Images were quantified for the plasma membrane localisation of S1PR1-GFP as in (B). Data are mean ± SEM of 3 independent experiments. Statistics are one-way ANOVA

with Tukey's multiple comparisons correction; black p-values denote significant differences, grey p-values are not significant.

Supplemental Figure 3. P-Rex1 limits the S1P-dependent internalisation of S1PR1 independently of its catalytic Rac-GEF activity. (A) Schematic showing quantification of receptor localisation at the plasma membrane by Volocity or CellProfiler image analysis. Mask 1 is generated to cover the entire cell and shrunk inwards by 0.619 µm (3 pixels) to generate mask 2. The fluorescent signal at the cell edge (mask 1 minus mask 2) is calculated as % of the total. **(B, C)** Expression of wild type or GEF-dead P-Rex1 limits the S1P-induced internalisation of S1PR1 in HEK293-S1PR1 cells. HEK293-S1PR1 cells were transfected with wild type (B) or GEF-dead (C) EE-P-Rex1 (blue symbols), or mock transfected (grey symbols), serum-starved for 6.5 h, and stimulated with 10 nM S1P for 0 or 30 min, fixed and stained with EE antibody. The amount of S1PR1-GFP at the plasma membrane was quantified by Volocity image analysis as in (A). Data are mean ± SEM of 3 independent experiments, the same as those shown in Figures 1A and 1B. Statistics are two-way ANOVA with Sidak's multiple comparisons correction; p-values in black are significant, p-values in grey are not. **(D)** P-Rex1 does not affect total S1PR1-GFP levels. Total lysates of HEK293-S1PR1 cells expressing EE-P-Rex1, or mock-transfected, were western blotted with P-Rex1 and GFP antibodies. Coomassie staining was used to control for protein loading. Western blots were quantified by Fiji densitometry. Data are mean ± SEM of 3 independent experiments. Statistics are paired t-test; p-values in grey are not significant.

Supplemental Figure 4. P-Rex1 deficiency does not affect EFGR localisation in PC12-S1PR1 cells. (A-C) Validation of cell fractionation method. PC12-S1PR1 cells were serum-starved for 14 h, stimulated with 5 nM S1P for 10 min, or mock-stimulated, and homogenised by douncing in detergent-free homogenisation buffer. Samples were centrifuged at 800 × g for 10 min and OptiPrep was added to the post-nuclear supernatant to 10 %. Samples were loaded onto a discontinuous OptiPrep gradient as in (A) and ultracentrifuged for 18 h at 90,000 × g. Fractions were collected from each interphase and proteins precipitated using TCA, washed in acetone, and analysed by western blotting using K-Ras as plasma membrane marker, EEA1 as early endosome marker, and Rab5 as endosome marker. All of fractions 1-5 and 50% of fraction 6 were loaded. Coomassie was used to show total protein. (B) Quantification of western blots by Fiji densitometry. Data are mean ± SEM of 4 independent experiments. (C) Western blots from one experiment representative of the data shown in (B). (D) P-Rex1 deficiency does not affect EGFR localisation. *Prex1*$^{+/+}$ (grey) and *Prex1*$^{-/-}$ (blue) PC12-S1PR1 cells were treated as described here-above and western blotted with EGFR antibody. Lower left: Endogenous EGFR was quantified by Fiji

densitometry. Lower right: no change in EGFR localisation in fractions 1 and 2 upon S1P stimulation. Data are mean ± SEM of 5-6 independent experiments, the same set as shown for S1PR1 in Figure 2D. Statistics are two-way ANOVA with Sidak's multiple comparisons correction and revealed no differences between genotypes.

Supplemental Figure 5. The DEP, PDZ and IP4P domains of P-Rex1 are required for the inhibition of S1PR1 internalisation. (A) P-Rex1 mutant expression. HEK293-S1PR1 cells were transiently transfected to express wild type EE-P-Rex1 (WT), GEF-dead EE-P-Rex1 (GD), EE-P-Rex1 ΔPH, EE-P-Rex1 ΔDEP, or EE-P-Rex1 ΔPDZ, or were mock-transfected, and total cell lysates were prepared and western blotted with P-Rex1 antibody and with tubulin antibody as loading control. **(B-E)** Volocity analysis of S1PR1-GFP localisation in P-Rex1 mutant expressing cells. HEK293-S1PR1 cells were transfected to express (B) EE-P P-Rex1 ΔPH, (C) EE-P P-Rex1 ΔDEP, (D) EE-P P-Rex1 ΔPDZ, or (E) EE-P P-Rex1 ΔIP4P (blue symbols), or were mock-transfected (grey symbols), serum-starved for 6.5 h, and stimulated with 10 nM S1P for 0 or 30 min, fixed and stained for EE. The amount of S1PR1-GFP at the plasma membrane was quantified by Volocity image analysis (see Supplemental Figure 3A). Data are mean ± SEM of 3 independent experiments for each mutant, the same experiments as shown in Figure 3; statistics are two-way ANOVA with Sidak's multiple comparisons test; black p-values denote significant differences, grey p-values are not significant.

Supplemental Figure 6. The isolated PDZ domain tandem of P-Rex1 is not sufficient to control GPCR trafficking, but P-Rex2 plays a similar role to P-Rex1. (A) The isolated PDZ domain tandem of P-Rex1 (iPDZ) is not sufficient to control S1PR1 internalisation. HEK293-S1PR1 cells were transfected with the myc-tagged iPDZ of P-Rex1 (blue symbols), or mock transfected (grey symbols), serum-starved for 6.5 h, stimulated with 10 nM S1P for the indicated periods of time, fixed, and stained with myc antibody. Top: Representative images of cells stimulated for 0 or 30 min. Middle: Quantification of S1PR1-GFP at the plasma membrane by comparison to standard images (see Supplemental Figure 1). Bottom: Quantification by Volocity image analysis (see Supplemental Figure 2A). Data are mean ± SEM of 3 independent experiments. Statistics are two-way ANOVA with Sidak's multiple comparisons correction; they showed no difference. **(B)** P-Rex2 inhibits the S1P-stimulated internalisation of S1PR1-GFP. HEK293-S1PR1 cells were treated as in (A) except that wild type myc-P-Rex2 (blue) was expressed. Quantification of S1PR1-GFP localisation was done as in (A). Data are mean ± SEM of 3 independent experiments. Statistics are two-way ANOVA with Sidak's multiple comparisons correction; stars denote differences between conditions with and without P-Rex2 for each time point; * indicates $p < 0.05$, ** $p < 0.01$, *** $p < 0.001$, and **** $p < 0.0001$.

Supplemental Figure 7. P-Rex1 does not control the agonist-induced internalisation of the RTKs EGFR and PDGFR. (A) EGFR. EGFR-GFP was expressed in MDCK cells with dox-inducible expression of wild type (circles) or GEF-dead (triangles) P-Rex1. Cells were treated with 1 µg/ml dox (blue symbols) for 24 h, or mock-treated (grey symbols), serum starved and then stimulated with 100 ng/ml EGF for the indicated periods of time, fixed, stained with Hoechst 33342, and imaged by confocal fluorescence imaging with focus on a central plane. Representative confocal images are shown. EGFR-GFP localisation was quantified by comparison to standard images (see Supplemental Figure 1). **(B)** PDGFR. MDCK cells were treated as in (A) except that PDGFRβ-GFP was expressed, and cells were stimulated with 40 ng/ml PDGF. PDGFRβ-GFP localisation was quantified as in (A). Data in (A, B) are mean ± SEM of three independent experiments for each receptor. Statistics are two-way ANOVA with Sidak's multiple comparisons correction.

Supplemental Figure 8. P-Rex1 does not interact with S1PR1-GFP, binds Grk2 directly, and interacts with full-length Grk2 but not the isolated catalytic domain of Grk2 in HEK293-S1PR1 cells. (A) HEK293-S1PR1 cells were transfected with myc-P-Rex1, or mock-transfected, serum-starved for 14 h, stimulated with 100 nM S1P, or mock-stimulated, for 10 min, lysed, subjected to immunoprecipitation (IP) with GFP antibody, and analysed by western blotting with myc and S1PR1 antibodies. 1.5% of the total lysate (TL) and IP supernatant (sup) and all the IP sample were loaded. Coomassie staining was used as a loading control. Blots shown are representative of three independent experiments. **(B)** P-Rex1 binds directly to Grk2 *in vitro*. Myc-P-Rex1 was incubated with GST or GST-Grk2, the GST-containing proteins were isolated using GSH-beads, and samples were western blotted with P-Rex1 and GST antibodies and quantified using Fiji densitometry. Data are mean ± SEM of three independent experiments; statistics are paired t-test. Representative blots from one of these experiments are shown in Figure 6B. **(C)** P-Rex1 interacts with Grk2 but not with the isolated catalytic domain of Grk2 *in vivo*. HEK293-S1PR1 cells expressing myc-P-Rex1 alone or with full-length flag-Grk2 (FL) or the flag-tagged isolated catalytic domain of Grk2 (iCAT), were serum-starved for 14 h, and total lysates were subjected to immunoprecipitation (IP) with flag antibody and analysed by western blotting with myc and flag antibodies. 1.5% of the total lysate (TL) and IP supernatant (sup) were loaded alongside all the IP sample (red boxes). Representative western blots are shown. Blots were quantified by Fiji densitometry. Data are mean ± range of 2 independent experiments. Statistics are one-way ANOVA with Tukey's multiple comparisons correction; black p-values denote significant differences, grey p-values are not significant.

Supplemental Figure 9. P-Rex2 binds Grk2 directly, independently of its Rac-GEF activity. (A) Human recombinant wild type His-P-Rex2 (blue bars) and mutated His-P-Rex2^{E30A,N212A} (GEF-dead) were produced

in Sf9 cells and purified using Ni-NTA agarose. A coomassie-stained gel of the purified proteins is shown. To confirm that wild type His-P-Rex2 is active and GEF-dead His-P-Rex2 is not, the purified proteins were tested in a liposome-based *in vitro* GEF activity assay, using GDP-loaded EE-Rac1 as the substrate and the indicated doses of PIP_3 to activate P-Rex2. Rac-GEF activity (^{35}S-GTPγS loading of EE-Rac1) is quantified as % of a positive control containing EDTA. Data are mean ± SEM of 3 independent experiments; statistics are two-way ANOVA with Sidak's multiple comparisons correction; stars denote differences for each PIP_3 concentration; ** indicates $p < 0.01$. **(B)** Binding assay. Wild type and GEF-dead His-P-Rex2 proteins as in (A) were incubated with GST or GST-Grk2, the GST-containing proteins were isolated using GSH-beads, and samples were western blotted with P-Rex2 and GST antibodies. 10% of the reaction mix (RM) and pull-down supernatant (sup) controls and all the pull down (PD) sample were loaded. Blots are representative of 3 independent experiments.

Supplemental Figure 10. Gβγ proteins partially compete out the binding of EE-P-Rex1 to GST-Grk2, but only at excess molar ratio. 5 pmol recombinant EE-P-Rex1 protein were incubated with 5 pmol GST-Grk2 and increasing concentrations (0, 2, 5, 20 pmol) EE-$G\beta_1\gamma_2$ proteins, the GST-containing proteins were isolated using GSH beads, and samples were analysed by western blotting using P-Rex1, EE, and GST antibodies. (A) Blots from 1 representative experiment of 3 are shown. (B) Quantification of P-Rex1 in pull down samples by Fiji densitometry. Data are mean ± SEM of 3 independent experiments. Statistics are one-way ANOVA with Tukey's multiple comparisons correction; black p-values denote significant differences, grey p-values are not significant.
